# Seasonal Dynamics of Fruit Flies (Diptera: Drosophilidae) in Natural Parks of Moscow City, Russia

**DOI:** 10.3390/insects15060398

**Published:** 2024-05-29

**Authors:** Nicolay G. Gornostaev, Yulia V. Lyupina, Oleg E. Lazebny, Alex M. Kulikov

**Affiliations:** N.K. Koltzov Institute of Developmental Biology RAS, 119334 Moscow, Russia; n_gornostaev@mail.ru (N.G.G.); yulial@bk.ru (Y.V.L.); amkulikov@gmail.com (A.M.K.)

**Keywords:** species diversity, fauna, abundance, ecology, seasonal fluctuation

## Abstract

**Simple Summary:**

This study represents the first investigation of the seasonal dynamics and species diversity of Drosophilidae in Moscow, Russia, from 2021 to 2023. Traps were placed on the ground under trees to collect the specimens. Among the collected species, *Drosophila obscura* Fll., *D. phalerata* Mg., and *D. testacea* Roser were the most abundant. Peaks in the abundance of drosophilids varied between years, but the lowest abundance was always observed in May. In 2022, the highest number of flies was collected (9604 specimens), and the highest species diversity (33 species) was recorded. The effects of temperature and precipitation on the species abundance and community diversity indices are evident during the preimaginal developmental stages of drosophila.

**Abstract:**

The insect fauna of natural parks in large cities has not been sufficiently studied in Russia. This study represents the first investigation of the seasonal dynamics and species diversity of Drosophilidae in Moscow city. Traps with fermenting liquid were placed on the ground under trees to collect flies from four natural park sites between early May and late September from 2021 to 2023. A total of 26,420 individuals belonging to 11 genera and 33 drosophilid species were identified, with 21 species from 6 genera being new to the fauna of Moscow. *Drosophila obscura* Fll., *D. phalerata* Mg., and *D. testacea* Roser were the most abundant species in the traps. Peaks in the abundance of drosophilids varied between years, but the lowest abundance was always observed in May. In 2022, the highest number of flies was collected (9604 specimens), with slightly fewer in 2023 (8496 specimens), and even fewer in 2021 (8320 specimens). In 2022, the highest species diversity of drosophilids was also recorded—33 species—while 28 species were found in both 2021 and 2023. The high variability in the abundance of individual drosophila species obscures the differences between the studied years due to the effects of the “Month” and “Site” factors. The diversity metrics exhibit similar patterns among drosophila communities inhabiting comparable biotopes. Specific climatic factors, such as the temperature and precipitation, impact the species abundance and community diversity indices primarily through their effects on the preimaginal stages of drosophila development. For several species, the population dynamics in the spring, post-hibernation, are influenced by the conditions preceding winter.

## 1. Introduction

The issues of urbanization and ecology in human settlements, especially in large cities, have garnered increased attention [1]. In comparison to rural areas, urban environments typically exhibit lower species diversity. However, insects, with their small size and ability to fly, thrive more easily in the natural parks of large cities.

Consequently, studying insect diversity in urban parks and green spaces is of particular interest [1]. For instance, research on bee species diversity in New York City gardens identified 54 species [2], while a suburban study found 110 bee species, suggesting that suburban landscapes are conducive to diverse bee populations [3]. Similarly, butterfly diversity along an urban gradient in California revealed that moderately disturbed sites harbored the highest species diversity, whereas species abundance decreased from natural to urban sites [4]. A similar study in Canada found that moderately disturbed sites hosted the highest abundance and species richness of butterflies, with species classified as disturbance adaptable and as disturbance avoiders [5].

Drosophilidae, commonly known as fruit flies, are renowned as model organisms in various biological fields [6,7,8,9,10,11]. Certain drosophilid species have adapted well to urban environments, displaying a synanthropic lifestyle and spreading alongside humans. With over 4600 species worldwide, Drosophilidae rank among the most ecologically diverse families within the order Diptera. Their larvae feed on fermenting and decaying substrates, including fruits, tree sap, mushrooms, living flowers and leaves, and even serve as predators [12,13,14,15,16,17,18].

Numerous studies have explored the fauna and ecology of Drosophilidae in urban areas globally, including Brazil [19,20,21,22], Uruguay [23,24], the USA [25,26,27], Ireland [28], the Netherlands [29], France [30], South Africa [31,32], Japan [33,34], and others. In Russia, some studies have investigated the fauna and ecology of drosophilids in urban areas [35,36,37,38,39], but research on their abundance and species richness fluctuations in natural city parks is lacking.

In the Central European part of Russia, anthropogenic pressures on wild ecosystems are substantial. Yet, insect assemblages in temperate zones exhibit resilience and adaptation capabilities [40,41,42,43]. Thus, it is particularly intriguing to examine drosophilid species richness and abundance fluctuations in the natural parks of a major city.

This study aims to investigate, for the first time, the seasonal dynamics and species diversity of drosophilids in Moscow’s natural parks, the largest city in Russia. Four natural parks were selected, each with four collection sites, characterized by varying dominant tree compositions.

## 2. Materials and Methods

### 2.1. Study Area

The study was carried out in Moscow, Russia, located in the center of European Russia (56°01′–55°08′ N, 36°48′–37°58′ E; up to 255 m a.s.l.). Moscow boasts numerous natural parks that contribute to its insect biodiversity. The total area of Moscow city is 2561 km^2^, with parks and forests covering from 0.5 to 85% of the area depending on the Moscow District. The mean annual precipitation is 550–650 mm, with a temperate climate characterized by an average annual air temperature of about +5.8 °C. The average temperature of the coldest month, January, hovers around −7.0 °C, with occasional drops to as low as −30.8 °C in recent years. Conversely, July, the warmest month, records an average temperature of about +24 °C, but extremes can reach up to +34 °C. Moscow experiences a humid continental climate characterized by warm or hot summers and long, cold winters. Summer typically lasts from mid-May to early September, while winter spans from early November to late March.

The flies were collected from the beginning of May to the end of September in 2021–2023 from four sites in four natural parks within Moscow city (Figure S1). The first site (No. 1) was situated in a birch biotope within the Bitsevsky Forest Natural Historical Park, the third largest park in Moscow, spanning approximately 2208 hectares. Each trap was positioned over 1 km away from the nearest city building (55°37′ N, 37°33′ E). The second site (No. 2) was located in a mixed deciduous biotope among birch, maple, rowan, and ash trees within Fili Park, covering about 266 hectares. The distance from the trap to the nearest city buildings was approximately 500 m (55°74′ N, 37°46′ E). The third site (No. 3) was positioned in a mixed deciduous biotope among birch, maple, linden, and ash trees within the Main Botanical Garden, covering about 331 hectares. The distance from the trap to the nearest city buildings was approximately 800 m (55°74′ N, 37°46′ E). The fourth site (No. 4) was located in a ravine among old linden trees, oaks, maples, and ash trees in Suvorovsky Park, a moderate sized park in Moscow covering about 280 hectares. The distance from the trap to the nearest city buildings was approximately 400 m (55°44′ N, 37°26′ E). All trap sites were at least 300 m away from paved surfaces.

### 2.2. Sampling

Standard plastic 5 L water containers with a cut-out window on one side positioned 10 cm above the bottom [44] were used as traps, with two traps per site placed on the ground and tied to the nearest tree trunk approximately 5 m apart. To attract flies, a mixture of beer, honey, and sugar was used as bait. The samples were collected once a week, and the bait was refreshed each time. Sampling was conducted by N.G. Gornostaev and Y.V. Lyupina. The collected samples were washed, immersed in alcohol, and transported to the laboratory.

### 2.3. Identification and Statistical Analysis

Drosophilid identification was performed by N.G. Gornostaev using a drosophilid key [45], with the species classified based on D. Grimaldi’s interpretation [46]. The species new to Moscow fauna are marked with an asterisk. The statistical analysis, supervised by A.M. Kulikov and O.E. Lazebny, included constructing distribution diagrams using Excel software LTSC MSO 64, employing nonparametric median and Kruskal–Wallis H tests to validate factors like “month” and “site”. The Categorical Principal Components Analysis (CATPCA) was used to visualize the relationship between species abundance, month, and biotope. Nested MANOVAs were performed using Statistica 64 software (Stat Soft. Inc., Tulsa, OK, USA). Ecological indices to characterize the assemblage, such as the diversity (the Shannon-Weiner Species Diversity Index), relative abundance, dominance, equitability, and constancy, were calculated.

## 3. Results

### 3.1. Faunal Composition

The trapping efforts in the natural parks in Moscow city yielded specimens from 5 genera and 8 species of the subfamily Steganinae along with 6 genera and 25 species of the subfamily Drosophilinae.

Notably, our survey identified 21 new species in 9 genera in Moscow’s fauna, including *Amiota albilabris*, *A. alboguttata*, *A. subtusradiata*, *Gitona distigma*, *Leucophenga maculata*, *L. quinquemaculata*, *Phortica semivirgo*, *Stegana coleoptrata*, *Chymomyza amoena*, *C. caudatula*, *C. costata*, *C. fuscimana*, *Drosophila busckii*, *D. funebris*, *D. histrio*, *D. hydei*, *D. kuntzei*, *D. melanogaster*, *D. subobscura*, *Hirtodrosophila* confusa, and *Scaptomyza graminum* (Table 1).

### 3.2. Abundance and Seasonal Dynamics of Drosophilidae

A total of 26,420 individuals belonging to 11 genera and 33 drosophilid species were identified in the traps set across four natural parks in Moscow between early May and late September from 2021 to 2023. The highest number of flies was collected in 2022 (9604 specimens), with slightly fewer in 2023 (8496 specimens) and even fewer in 2021 (8320 specimens) (Table 2).

Fifteen drosophilid species (*D. obscura* (11,103), *D. testacea* (3903), *D. phalerata* (3680), *D. melanogaster* (1768), *S. rufifrons* (940), *D. histrio* (821), *D. repleta* (786), *D. kuntzei* (619), *D. transversa* (552), *D. subsilvestris* (471), *P. semivirgo* (324), *D. immigrans* (309), *L. quinquemaculata* (271), *D. busckii* (228), and *M. poecilogastra* (201)) were found in quantities exceeding 100 individuals collectively over the three years, making them the most common species. Notably, *D. obscura*, *D. phalerata*, and *D. testacea* were classified as mass species, with a combined total number exceeding 1000 individuals per year (Table 2). The remaining 18 species, collected in quantities ranging from 1 to 90 flies over the three years, were categorized as rare and extremely rare in the natural parks. This group mainly includes drosophilids that are less attracted to traps with fermenting bait (species of Amiota, Chymomyza, Gitona, Stegana, Hirtodrosophila, and Scaptomyza).

**Figure 1:**
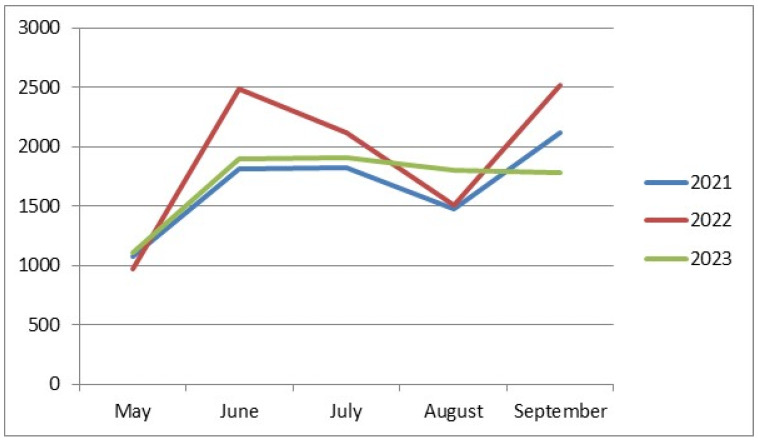
Seasonal dynamics of drosophilid abundance depending on the year of collection. On the abscissa axis—the time of collection; on the ordinate axis—the number of individuals.

The lowest abundance of drosophilids was recorded in May (1076, 968, and 1113 individuals in 2021–2023, respectively) (Figure 1). This is the only similarity among all our collections over all three years. The rest of the data on drosophilid abundance noticeably differ from year to year. In 2021, we observed an increase in the drosophilid numbers in June, a slight further increase in July, a decrease in August, and a sharp rise to the annual maximum abundance in September (1812, 1829, 1478, and 2125 individuals from June to September, respectively). In 2022, we observed two peaks in drosophilid abundance in June and September, with decreases in July and August (2490, 2121, 1507, and 2518 individuals from June to September, respectively). Finally, in 2023, there was an increase in drosophilid abundance in June, followed by a plateau in July, then a very gradual decrease in abundance in August and September (1897, 1905, 1801, and 1780 individuals from June to September, respectively).

This suggests that the minimal abundance of drosophilids in May may be associated with their pupal stage after overwintering and their gradual emergence during this period. The variation in the seasonal dynamics in other months could be related to the climatic peculiarities of each year of the study.

### 3.3. Climate Characteristics during the Collection Period of Drosophilids in 2021–2023

The years during which Drosophilids were collected in Moscow parks noticeably differed in climatic conditions (Table 3). The most interesting differences were observed during the summer of 2022. In June and July, the average monthly temperature exceeded the norm by 2.2 °C and 2.5 °C, respectively, while in August, it exceeded by 5.5 °C. Additionally, the summer of 2022 was marked by drought, with only July reaching the precipitation norm, while June received less than half of the norm, and August saw only 5% of the norm. However, it was in 2022 that the highest number of Drosophilids was collected in traps, accompanied by the highest species diversity. It can be hypothesized that the climate influences Drosophilid populations in the temperate zone where our research was conducted, but further extensive studies are required for a more precise assessment of this influence.

### 3.4. Species Diversity of Drosophilidae

To assess the species diversity within communities, the Shannon–Wiener diversity index and the Simpson dominance index are primarily used.

The Shannon–Wiener diversity index attains its maximum value when the distributions of all species in the sample is perfectly even. In natural communities, this signifies an equal abundance of all species in the community. The minimum value of the Shannon index is reached when the entire community is represented by a single species. The Simpson dominance index evaluates the probability that two individuals randomly selected from the community belong to the same species; it indicates the proportion of dominant species in the community.

**Figure 2:**
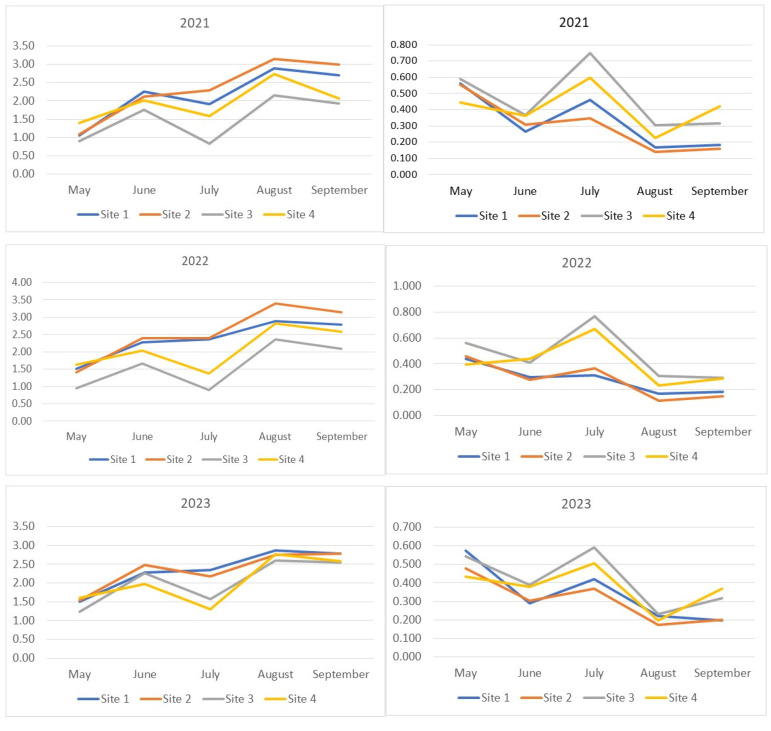
Seasonal dynamics of diversity indices (Shannon–Wiener) and Simpson dominance for each year of observation. The x-axis represents the collection months, while the y-axis shows the index values. On the left, the seasonal changes in the Shannon–Wiener index for 2021–2023 are displayed, while on the right, the Simpson dominance indices are shown.

In our study, the increase in Drosophilidae diversity was accompanied by an increase in the number of rare species, while the decrease was associated with an increase in the proportion of dominant species. In the middle of the summer, the diversity decreased to levels characteristic of the spring period (Figure 2). This trend was least pronounced in the Drosophilidae communities of the Bitsevsky and Fili parks, where the absence of diversity reduction or even a slight increase was accompanied by an increase in the dominance index, indicating a decrease in the relative abundance of rare species. The maximum diversity was observed in August. In 2021 and 2023, the Main Botanical Garden exhibited the lowest community diversity of Drosophilidae, while Fili Park showed the highest diversity from the second half of the summer onwards. In 2022, despite the highest number of recorded species, the increase in the diversity index was accompanied by an increase in the dominance index, indicating a decrease in the overall proportion of rare species and an equalization of the abundance of common species. The Fili and Bitsevsky parks showed the highest diversity, while the Main Botanical Garden exhibited the lowest diversity (Figure 2).

Interesting patterns can be derived from the analysis of the seasonal and annual dynamics of the Shannon–Wiener indices of samples grouped by drosophilid sampling location (Supplementary Materials, Figure S2a–d). Drosophilid communities are paired based on the curve positioning, reflecting index dynamics over the years. For Bitsevsky Park (Site 1) and Fili Park (Site 2), the Shannon–Wiener index values obtained for the year 2022 are maximal for three (Site 1) or all (Site 2) sampling months, while the values obtained for 2023, on the contrary, are mostly reduced. For the Main Botanical Garden (Site 3) and Suvorovsky Park (Site 4), the opposite pattern is observed. Interestingly, the highest values and the steepest increase in indices were observed for the Fili Park community (Site 2), while the lowest values and the most pronounced dynamics fluctuations were characteristic of the Main Botanical Garden community (Site 3).

**Figure 3:**
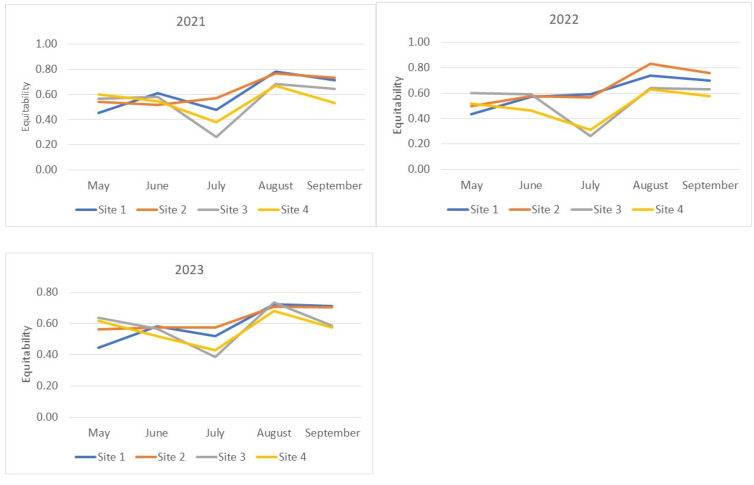
Evenness of species abundance distribution in the analyzed samples. Equitability (J’) of the fly community structure was calculated using Pielou’s Index formula: J’ = H’/log s, where J’ = Equitability measure, H’ = Shannon–Wiener value, and s = total number of species sampled.

The patterns of Equitability index changes (Figure 3) generally mirror those presented above for the Shannon–Wiener index. Some differences are observed in the relative changes of index values during the May–June period of 2021–2023 for the Suvorosky Park and Main Botanical Garden sites and during May–June 2021 for the Fili Park site. Noticeable increases in the diversity indices during this period are accompanied by either no growth or a decrease in the Equitability index. This outcome suggests an increase in the relative abundance of rare species in the samples from June compared to May. The lowest Equitability values, indicating the lowest evenness of species abundance in the samples, were observed for the Suvorosky Park and Main Botanical Garden sites compared to the other two collection points from July to September. An exception was noted in 2023, where in August, the Equitability index values for all sites reached a similar maximum level.

### 3.5. Relative Abundance and Constancy of Drosophilid Species

**Figure 4:**
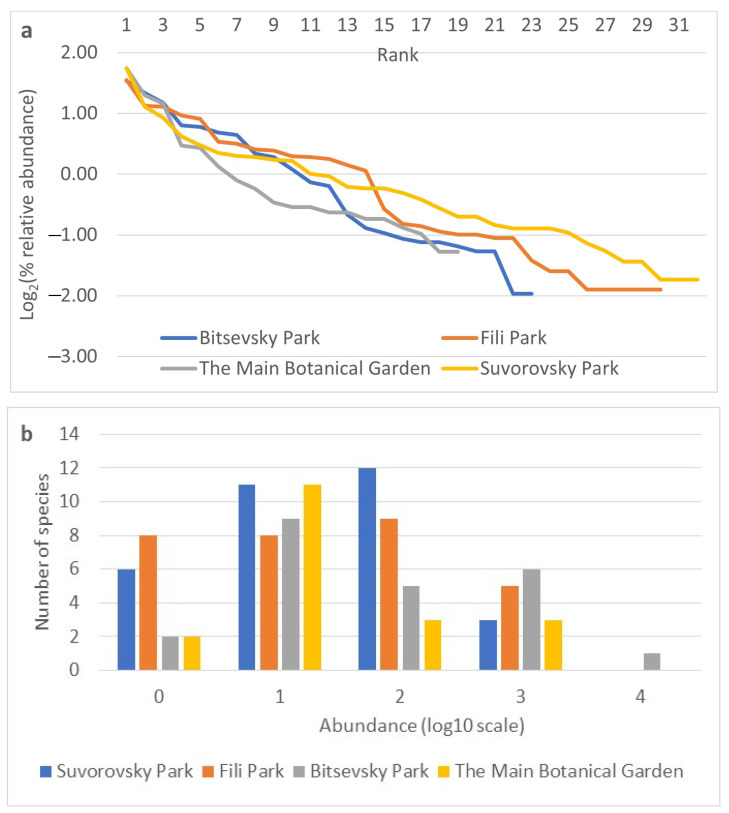
Relative abundance of species by collection sites. (**a**) Rank abundance diagram (Whittaker plot) of Drosophila samples from Moscow parks. Relative abundance is represented on a logarithmic scale to base 2. (**b**) Frequency histogram of species abundance distribution. Relative abundance is represented on a logarithmic scale to base 10.

The relative abundances of species indicators were obtained for each collection site based on data from all three years of observation (Figure 4a,b). The rank abundance curves for the studied sites are similar in sections characterizing dominant species (Figure 4a). Differences become more pronounced from the 4th to 5th rank, indicating a more rapid decline in the abundance of species at the Main Botanical Garden site from the 5th to the 10th rank, the Bitsevsky Park site from the 12th to the 14th rank, and the Fili Park site from the 14th to the 16th rank. The Fili Park site is characterized by the highest number of rare species. The distribution of species on the frequency histogram resembles a normal distribution (Figure 4b) when using the log10 scale of relative species abundance and rounding ranks to integer values. The Suvorosky Park and Fili Park sites exhibit a leftward shift in the distribution, towards rare species, while the Main Botanical Garden and Suvorosky Park sites show a rightward shift, towards dominant species.

The formal similarity and proximity of curves expressing the variability of indices characterizing different collection sites by year and season do not yet indicate significant similarities or differences in the observed fluctuations of these indices. To assess the significance of the observed variability in the species composition of Drosophilidae communities, a multivariate analysis of variance was conducted. This analysis used Hill numbers of order 1 and order *n* (^q=1^D; ^q=n^D), where *n* is the number of species in the sample, as the dependent variable. These numbers evaluate community diversity proportional to species abundance in the first case and predominantly abundant species in the community in the second. The independent factors included the month, temperature, precipitation, and the composition of the plant community.

The absolute temperature values vary significantly from month to month, and deviations from the average monthly temperatures may influence the life cycle of species and, consequently, the dynamics of their populations. To represent temperature and humidity as categorical variables, indicators of excess or deficiency of average monthly temperatures and precipitation were ranked. In both cases, five categories of values were obtained. The temperature regimen was represented by the norm (n) and deviations of minus 1 (m1) and minus 3 (m2), as well as plus 1 (p1) and plus 3 (p2). Here, −1 < n < 1, −3 < m1 ≤ −1, m2 ≤ −3, 1 ≤ p1 < 3, and p2 ≥ 3. Precipitation was represented as the norm (no), drought conditions as 1 and 2 (dry1 and dry2, respectively), and increased humidity conditions as 1 and 2 (wet1 and wet2, respectively). The norm ranged from 0.8 to 1.2 of the monthly precipitation norm (taking the norm as 100% or one unit). Deviation values for precipitation indicators corresponded to the following: 0.3 < dry1 ≤ 0.8, dry2 ≤ 0.3, 1.2 ≤ wet1 < 1.8, and wet2 ≥ 1.8.

The normality of the Hill numbers distribution was tested using the Kolmogorov–Smirnov test. It confirmed that the presented distributions do not significantly differ from normal (Hill numbers of order q = 1: K-S d = 0.09734, *p* > 0.20; Hill numbers of order q = n: K-S d = 0.14420, *p* < 0.20), and the variables can be used for a MANOVA.

The impact of deviations from climatic norms for this locality is superimposed on specific microclimate conditions, which vary across different plant communities, and on demographic conditions that are determined by the seasonality of the climate, changing during the spring, summer, and autumn periods. Accordingly, a hierarchical (“nested”) design for the distribution of factor levels was applied, and categories of temperature change norms and precipitation amounts were nested within the “Site” or “Month” factor levels. It is also important to consider the relatively short developmental period of Drosophila from egg to imago, ranging from two to four weeks depending on the temperature. Abundance assessments of the species were based on adult collections, while environmental conditions could influence not only the embryonic and larval stages but also the pupal stage and directly affect the imago. Therefore, correlations between the average monthly temperature and precipitation values and diversity assessments of Drosophilidae communities were conducted in two variants: based on the average monthly values of the preceding month and the month corresponding to the collections.

**Figure 5:**
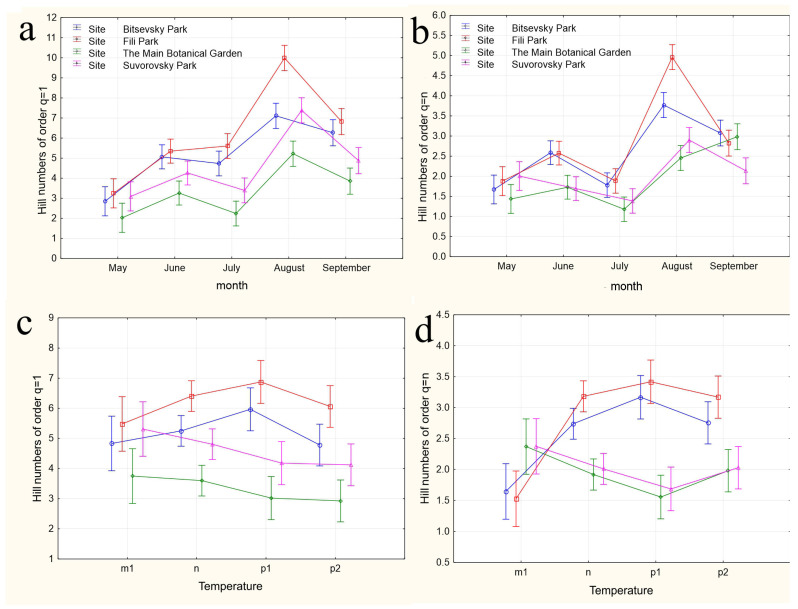

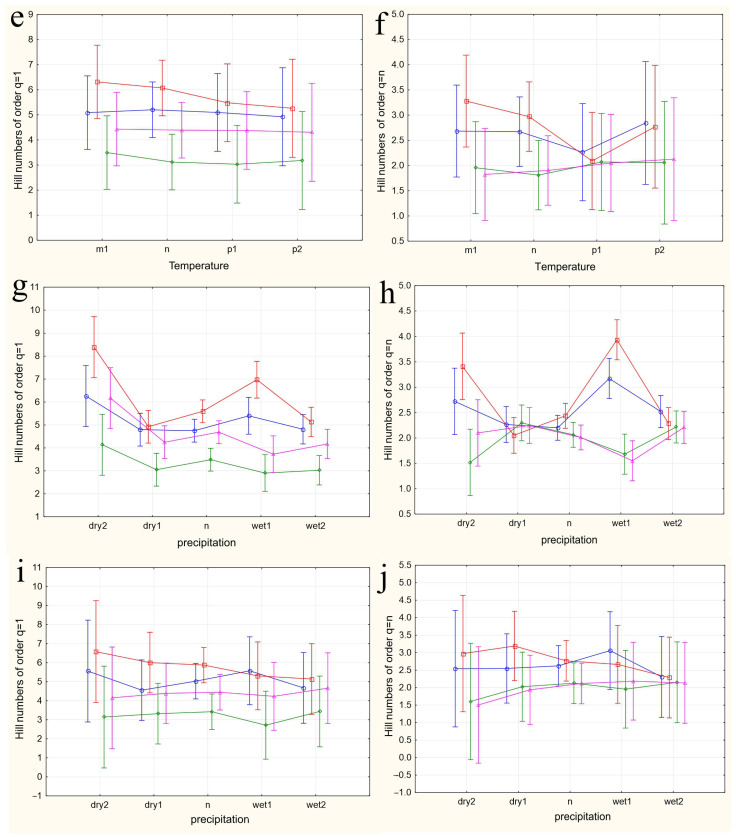

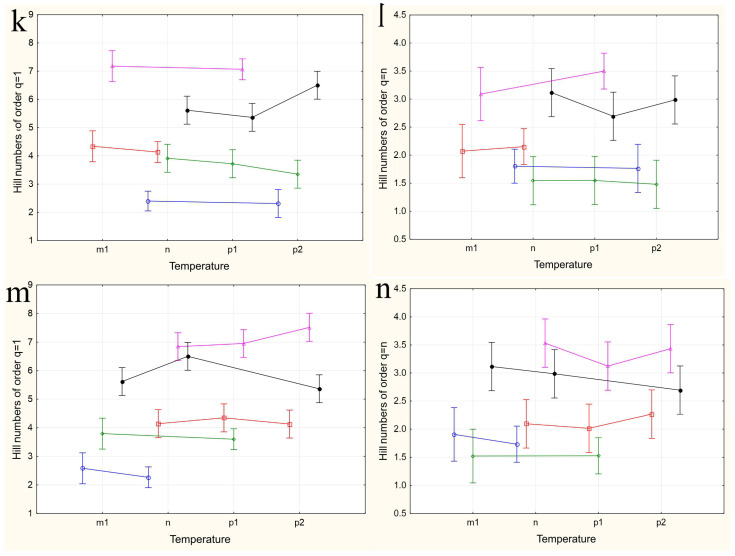
The variations in species diversity at sampling locations influenced by seasonal changes (**a**,**b**), deviations from the average monthly temperature (**c**–**f**,**k**–**n**), and precipitation norms (**g**–**j**). Type III decomposition: vertical bars denote 0.95 confidence intervals. (**a**) “Month” nested in “Site”, Hill n (q = 1). Wilks’s lambda = 0.00001, F(64, 37.509) = 11.689, and *p* = 0.00000; (**b**) “Month” nested in “Site”, Hill n (q = n). Wilks’s lambda = 0.00001, F(64, 37.509) = 11.689, and *p* = 0.00000; (**c**,**d**) Temperature indices correspond to the month preceding sampling. (**c**) “Temperature” nested in “Site”, Hill n (q = 1). Wilks’s lambda = 0.00064, F(48, 36.708) = 4.3903, and *p* = 0.00001. (**d**) “Temperature” nested in “Site”, Hill n (q = n). Wilks’s lambda = 0.00064, F(48, 36.708) = 4.3903, and *p* = 0.00001. (**e**,**f**) Temperature indices correspond to the month of sampling. (**e**) “Temperature” nested in “Site”, Hill n (q = 1). Wilks’s lambda = 0.56002, F(24, 22) = 0.30825, and *p* = 0.99695. (**f**) “Temperature” nested in “Site”, Hill n (q = n). Wilks’s lambda = 0.56002, F(24, 22) = 0.30825, and *p* = 0.99695. (**g**,**h**) Precipitation indices correspond to the month preceding sampling. (**g**) “Precipitation” nested in “Site”, Hill n (q = 1). Wilks’s lambda = 0.00021, F(64, 37.509) = 4.5064, and *p* = 0.00000, (**h**) “Precipitation” nested in “Site”, Hill n (q = n). Wilks’s lambda = 0.00021, F(64, 37.509) = 4.5064, and *p* = 0.00000. (**i**,**j**) Precipitation indices correspond to the month preceding sampling. (**i**) “Precipitation” nested in “Site”, Hill n (q = 1). Wilks’s lambda = 0.53971, F(32, 22) = 0.24832, and *p* = 0.99982. (**j**) “Precipitation” nested in “Site”, Hill n (q = n). Wilks’s lambda = 0.53971, F(32, 22) = 0.24832, and *p* = 0.99982. (**k**,**l**) Temperature indices correspond to the month preceding sampling. The legend indicates the month of temperature norm deviations assessment first, followed by the month of ^q^D diversity index evaluation. (**k**) “Temperature” nested in “Month”, Hill n (q = 1). Wilks’s lambda = 0.79738, F(2, 30) = 3.8116, and *p* = 0.03349. (**l**) “Temperature” nested in “Month”, Hill n (q = n). Wilks’s lambda = 0.79738, F(2, 30) = 3.8116, and *p* = 0.03349. (**m**,**n**) Temperature indices correspond to the month of sampling. (**m**) “Temperature” nested in “Month”, Hill n (q = 1). Wilks’s lambda = 0.47015, F(4, 58) = 6.6471, and *p* = 0.00018. (**n**) “Temperature” nested in “Month”, Hill n (q = n). Wilks’s lambda = 0.47015, F(4, 58) = 6.6471, and *p* = 0.00018.

The results of applying four variants of the MANOVA, considering two nested design options and two weather condition assessment methods, are presented in Table 4. Regardless of whether the “Site” or “Month” factor was used as the primary factor, both factors in all analysis variants showed high significance in influencing the differences in group mean diversity indices (^q^D). The significance of the nested factors “Temperature” and “Precipitation” was not confirmed when using the “Site” factor as the primary factor, and only the “Precipitation” factor was significant when using the “Month” factor as the primary factor. A visualization of the results of a more detailed one-way ANOVA, incorporating all factors considering the applied nested design, is provided in Figure 5a–n.

In the first case, a nested design MANOVA was used, where the effects of the month (season), temperature, and precipitation factors were considered within the Site factor. The figures depict changes in the species diversity at sampling points under the influence of the season (Figure 5a,b), deviations from the monthly temperature norm (Figure 5c–f,k–n), and precipitation norms (Figure 5g–j). The dynamics of diversity indices depending on the sampling month are best reflected in the graphs representing the categories “Month” nested in the factor “Site”. Formal pairwise similarities of the curves constructed using Hill numbers of order 1, characterizing the relationship between diversity and the factors “Site” and “Month”, are clearly visible for the communities of Bitsa Park and Fili Park in the first pair, and Suvorovsky Park and the Botanical Garden in the second. When comparing Hill numbers of order n, formal similarities change to significant differences within these pairs and become more pronounced. In other words, when considering abundant species, the similarity between parks in the noted pairs becomes much more evident. The relationship between the temperature and precipitation indices and species abundance is expressed through a more complex dependence (Figure 5c–n).

When using the “Site” factor as the main one and the nested factors “temperature” and “precipitation” values for the month preceding the analyzed sample (Figure 5c,d and Figure 5g,h, respectively), the dependence of diversity indices on different sites from nested factors is visible. The most pronounced dependence on temperatures is observed when considering numerous species (Hill numbers of order q = n, Figure 5d). The response of Drosophilidae communities inhabiting areas with mixed and predominantly deciduous forests (Bitsevsky Park and Fili Park) is reflected in an increase in the Shannon diversity index with a change in the temperature regimen from low to normal and high, and slightly decreases with extremely high average monthly temperatures. In habitats with predominantly broad-leaved tree species, we observe the opposite pattern of decreasing community diversity with increasing temperatures from low to high, and some increase in diversity when the temperature exceeds the threshold by 3 °C compared to the norm. Moreover, sites with similar habitats do not show significant differences in the diversity indices for most temperature regimens. Since the assessments were made based on the indicators of the months preceding each collection, the effect obtained is presumably associated with the impact of temperature on earlier stages of Drosophila development: from egg to pupa. It is indicative that the influence of different temperature regimens on diversity indices is not observed when using indicators of temperature deviations in months corresponding to collections (Figure 5e,f). The absence of significant changes in community diversity suggests the stability of imago populations and the composition of the entire community to temperature changes throughout the accounting period.

Similar results are observed when assessing the impact of precipitation on the diversity indicators. Here, the maximum effect is also noted when evaluating diversity based on the most representative species and using data from the months preceding the sampling (Hill numbers of order q = n, Figure 5h). In this case, normal precipitation levels or slight decreases, as well as significant increases up to maximum values, result in fluctuations of ^q=n^D around a median range of 2 < ^q=n^D < 2.5. Severe drought or moderate excess precipitation leads to an increase in diversity in habitats with mixed and predominantly small-leaved forests and a decrease in those with predominantly broad-leaved forests. The noted change in diversity with moderate excess precipitation was observed in May 2022 and August 2021 and preceded the summer and autumn population outbreaks, suggesting that the observed relationship may be indirect, and the precipitation levels themselves might not be the primary factor. Nonetheless, significant excess precipitation was also recorded in May 2021 and July 2023, corresponding to changes in the diversity indices back to a relative norm. This supports the hypothesis that the larval populations of the species composing the community are sensitive to external conditions. Again, the impact of changes in precipitation norms on diversity indices is not observed when using data corresponding to the month of sampling (Figure 5i,j). Presumably, the abundance indicators of the Drosophila community inhabiting urban parks are not sensitive to deviations from the monthly precipitation norms.

When using the “Month” factor as the main factor, the pattern of the influence of the nested factor “Temperature” on diversity indices ^q^D was obtained over three years of research. This corresponds to a maximum of three estimates, and the picture of changes in species diversity across the four categories of temperature deviations suffers from incompleteness (Figure 5k–n). Additionally, given the aforementioned opposite effects of temperature conditions on diversity indices at different sites, a high variability in the diversity indices and a lack of clear dependence of these indices for data aggregated by sites can be expected. Nevertheless, some dependencies are observed in this analysis variant as well. The dependence of diversity on temperature is confirmed for August and September and is significantly more pronounced for ^q=1^D indices, which consider rare and less abundant species. In the first analysis variant, using the values of temperature deviations for the month preceding the sampling time (Figure 5k,l), a significant increase in diversity in September is noted when August temperatures range from normal to maximum values. May temperatures ranged from below normal to normal, while June temperatures ranged from normal to maximum elevated values, and both diversity indices ^q=1^D (Figure 5k) and ^q=n^D (Figure 5l) significantly decreased. For abundant species, the ^q=n^D index (Figure 5l) increased in August as the July mean monthly temperatures rose from below normal to above normal. From these estimates, it can be concluded that in early summer, larvae negatively react to rising temperatures, decreasing diversity, while in late summer, they react positively, increasing it. The increasing variance in the diversity indices, associated with abundant species, masks the relationship between temperature and diversity at the end of summer.

When using the values of the nested factor “Temperature” for the month corresponding to the sampling time, temperature increases lead to opposite results. For instance, in August, an increase in the mean monthly temperatures leads to an increase in the ^q=1^D diversity index (Figure 5m), whereas in September, it leads to a decrease, which is more monotonic but with significantly higher variance in the ^q=n^D index estimates (Figure 5h,n). Changes in temperature norms from May to July do not have a significant impact on the diversity of the drosophilid community at the imago stage.

The species were grouped into six classes of constancy ranges and arranged from highest to lowest values: I. high constancy weed species (species that were recorded in all sites), II. moderately high constancy weed species (species that were recorded in 12–14 of the sites), III. intermediate constancy weed species (species that were recorded in 8–11 of the sites), IV. low constancy weed species (species that were recorded in 4–7 of the sites), V. rare constancy weed species (species that were recorded in 1–3 of the sites), and VI. very rare constancy weed species (species that were not recorded in this station but present in other stations) (Table 5).

When assessing the constancy values for the recorded species based on the combined data from all years and sampling seasons, the abundance of the species, as well as the collection site and seasonality, play a role. *D. obscura* is a mass species present in all collections at all sites. Similarly, for the Bitsevsky Park site, *D. testacea* is a mass species. This species, along with *D. phalerata*, *D. subsilvestris*, *S. rufifrons*, *L. quinquemaculata*, and *D. histrio*, are frequently and moderately encountered across all collection sites. Differences between the collection sites in the constancy estimates reveal pairwise similarities between the Suvorovsky Park–Fili Park and the Main Botanical Garden–Bitsevsky Park sites. Species such as *M. poecilogastra*, *P. semivirgo*, *D. immigrans*, *H. trivittata*, *D. busckii*, *D. repleta*, *D. subobscura*, *A. albilabris*, *L. maculata*, and *D. hydei*, which are frequent or moderately encountered at the Suvorovsky Park–Fili Park sites, become rare or completely absent in collections from the Main Botanical Garden–Bitsevsky Park sites. This indicates differences in the ecological conditions between the specified pairs of drosophila collection sites.

### 3.6. The Impact of “Year”, “Month”, and “Collection Site” Factors on Drosophilid Abundance

Out of the 33 collected species, 28 were included in the analysis of the impact of the “collection year”, “collection month”, and “collection site” factors on species abundance. An examination of the distribution of species abundance measures in samples grouped by collection sites (Supplementary Materials, Table S1), by collection years (Supplementary Materials, Table S2), and by collection months (Supplementary Materials, Table S3) revealed that the distribution of species abundance in the majority of samples did not adhere to normality. According to the results of the Lilliefors test, a significant deviation from a normal distribution of species abundance was observed in 79.8% of samples grouped by collection sites, in 87.8% of samples grouped by collection years, and in 70.4% of samples grouped by collection months. Based on these results, non-parametric analysis methods, specifically a Kruskal–Wallis ANOVA by rank and the median test, were employed to assess the significance of the influence of each of the three factors (ecological conditions, collection year, and collection month). The Benjamini and Hochberg corrected significance level was applied to account for multiple comparisons (Table 6).

For samples combined according to the values of the assessed factor, the influence of the collection year on species abundance is not confirmed for any species. The influence of sampling site conditions significantly affects 60.7% of species, and the influence of the collection month is significant for most species, except for *L. maculata* and *D. hydei*.

The absence of an effect of the “Year” factor on the total species abundance does not mean that this factor does not interact with others or does not exert influence in conjunction with the “Month” and “Site” factors. To test the interaction of the “Year” factor with the “Month” or “Site” factors of sample collection, an analysis of the dependence of species abundance in a given month or at a given collection site on the year of study was conducted (Table S6). In no case was a significant influence of the collection year on the relative species abundance confirmed, both by collection months and by sites.

The dependence of the abundance of each species in the collections for each year on the month of collection or collection site was also analyzed (Table 7, Supplementary Materials, Table S5).

Taking into account the correction for multiple comparisons, 14 out of 28 species show a dependency of abundance on the sampling month in at least one of the years of the study, with *p*-values ranging between 0.05 and 0.10 in the remaining years (Table 7). These results support the conclusion regarding the role of seasonal conditions in the fluctuations of the abundance of these drosophilid species. The analysis of the influence of sampling site on the abundance of drosophilid species, conducted for each year of the study, did not confirm a significant effect of ecological conditions, considering the correction for multiple comparisons (Supplementary Materials, Table S5). Such a discrepancy with the results of the analysis of data combined over all three years of the study (Table 4) may be attributed to the reduction in the number of analyzed samples in the data for individual years of the study and to a more pronounced effect of random seasonal fluctuations in species abundance, masking the influence of specific sampling site conditions.

**Figure 6:**
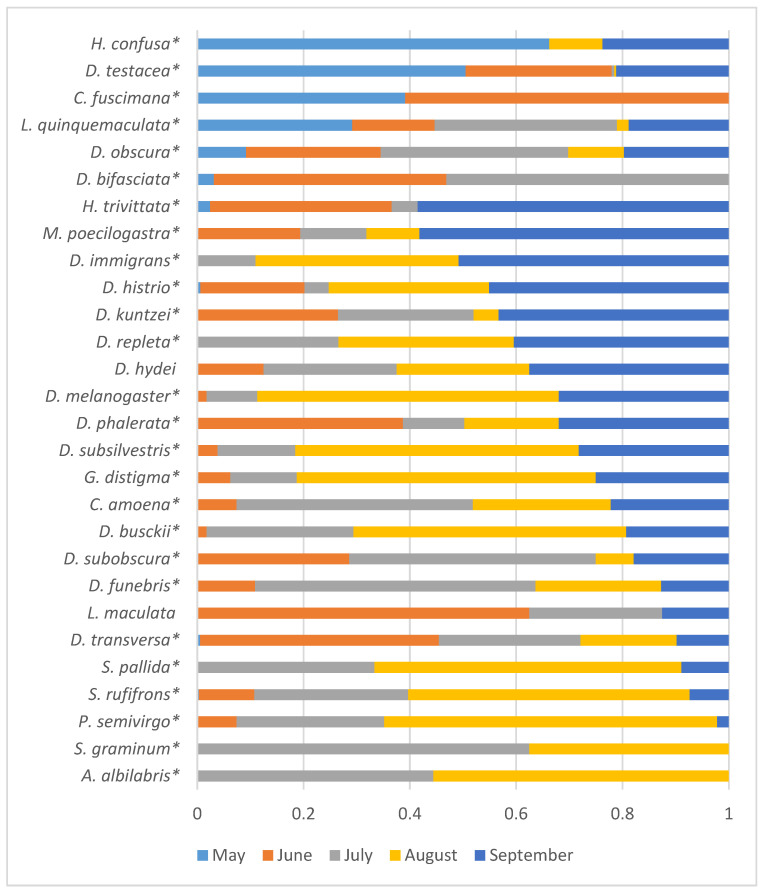
Distribution of species abundance by month of sampling, as proportions of the total abundance summed over all three years of the study. Species with significant influence on the abundance of the sampling month are marked with asterisks (Table 4).

Given the lack of significant influence of the year of sampling on the seasonal fluctuations in species abundance, the data are presented in Figure 6 as proportions of the total species abundance summed over the three years of the study.

Four Drosophilid species with maximum abundance were observed in May and May–June (*H. confusa*, *D. testacea*, *C. fuscimana*, and *L. quinquemaculata*). For ten species, the maximum abundance was recorded in August and September (*H. trivittata*, *M. poecilogastra*, *D. immigrans*, *D. histrio*, *D. repleta*, *D. hydei*, *D. melanogaster*, *D. subsilvestris*, *G. distigma*, and *D. busckii*).

**Figure 7:**
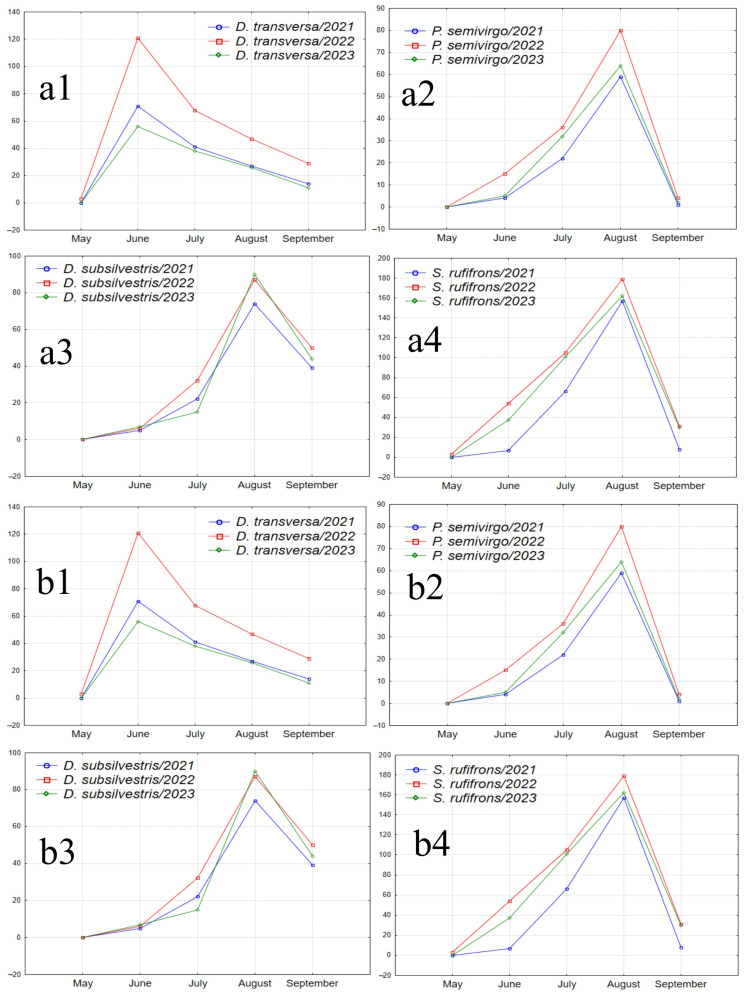

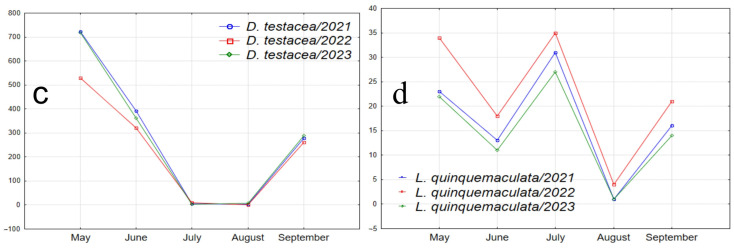
Graphs illustrating the seasonal abundance changes of species over the years of the study. (**a1**–**a4**) Species absent in May samples, with peak abundance in one of the summer months and a decline in September (*D. transversa*: peak abundance in June; *P. semivirgo*, *D. subsilvestris*, and *S. rufifrons*: peak abundance in August). (**b1**–**b4**) Species absent in May samples, with peak abundance in June or July, a decline in July or August, and a significant increase in abundance in September (*D. histrio*, *D. phalerata*, *D. obscura*, and *M. poecilogastra*). (**c**) Species with peak abundance in May, decreasing to near zero in summer months and increasing in September (*D. testacea*). (**d**) Species with peak abundance in May, an intermediate peak in mid-summer between two abundance declines, and an increase in September (*L. quinquemaculata*).

According to the analysis of the dependence of species abundance on the month of sampling for each year of the study (Table 4), it can be seen that in some cases, the obtained estimates can vary significantly across different years. For example, *G. distigma* and *D. subobscura* showed a significant dependence of abundance on the season only in 2022, while *S. pallida* did so in 2023. It is also interesting to assess the dynamics of seasonal abundance changes and identify weak effects that are not statistically confirmed for each individual species but are manifested by the majority of species. For this purpose, graphs illustrating the seasonal abundance changes of species over the years of the study are provided (Figure 7). To avoid distortions of the seasonal abundance dynamics due to random fluctuations, the analysis included species with a total abundance ranging from 200 to 11,103 individuals over the entire study period, and all synanthropic drosophilid species were excluded, as their presence in natural parks may depend on random food resources brought by humans.

**Figure 8:**
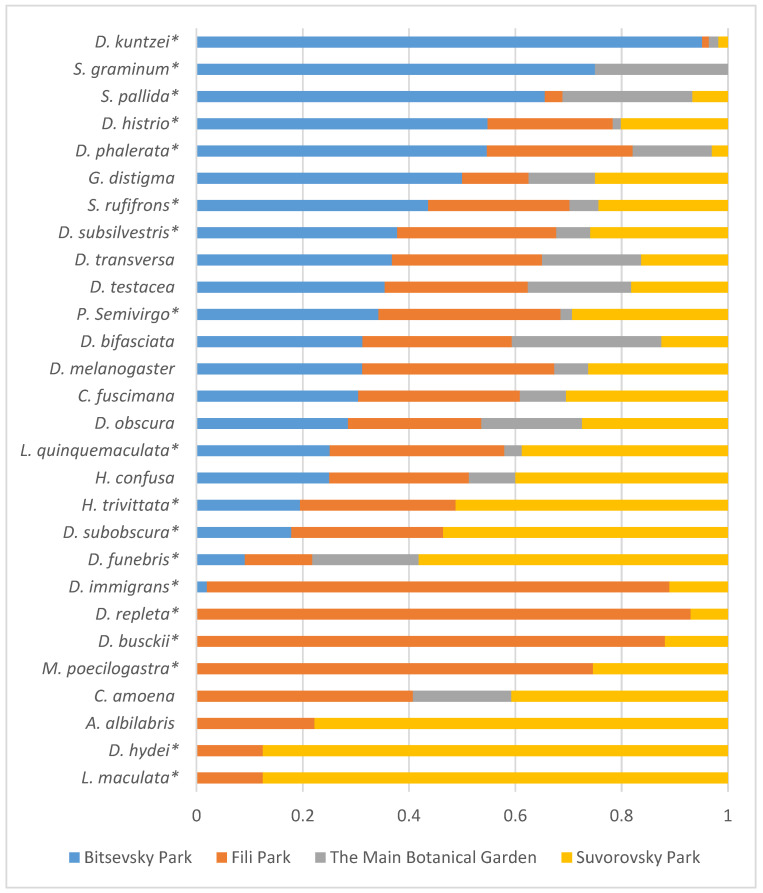
Distribution of relative species abundance (in %) across collection sites based on aggregated data from 2021–2023. Asterisks denote species with a significant impact on site abundance (Table 4).

Furthermore, Figure 8 illustrates the distribution of relative species abundance across collection sites. Flies in the samples were unevenly distributed among the sites. Among them, five species (*D. kuntzei*, *S. graminum*, *S. pallida*, *D. histrio*, and *D. phalerata*) were predominantly captured in Bitsa Park, while eight species (*D. immigrans*, *D. repleta*, *D. busckii*, *M. poecilogastra*, *C. amoena*, *A. albilabris*, *D. hydei*, and *L. maculata*) were found in Fili Park and Suvorovsky Park. Among the most evenly distributed between the collection sites were four species (*D. obscura*, *D. bifasciata*, *D. testacea*, and *D. transversa*) (Figure 8).

**Figure 9:**
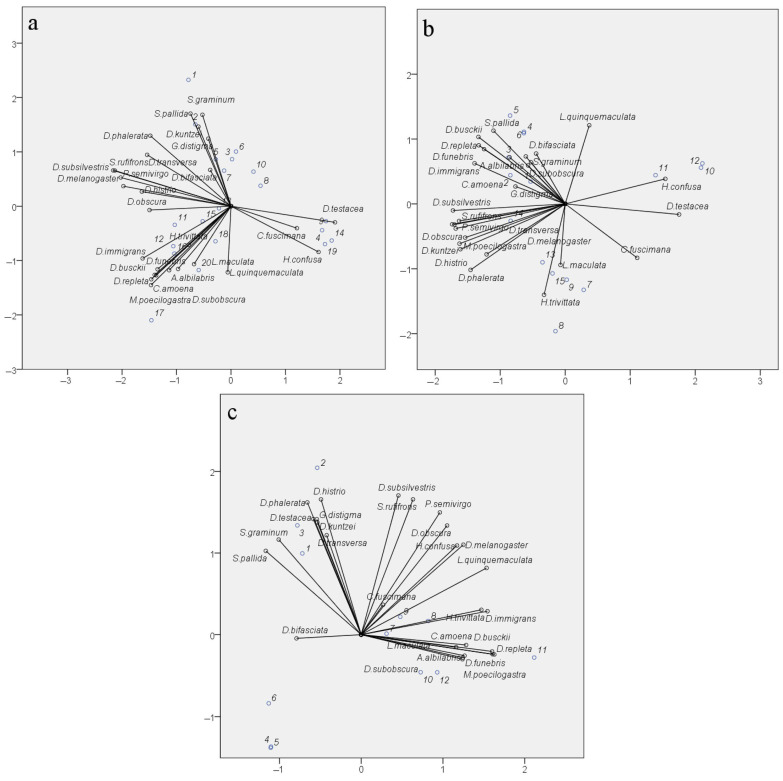
Categorical Principal Components Analysis (CATPCA) of drosophilid species distribution considering the conditions “Site”, “Month”, and “Year”. Bit_P—Bitsevsky Forest Natural Historical Park; Bot_G—Main Botanical Garden; Fili_P—Fili Park; Suv_P—Suvorovsky Park; x-axis—Principal Component 1; y-axis—Principal Component 2. (**a**) Species distribution by habitat considering collection month: numbers of objects on the biplot: 1—Bit_P_August; 2—Bit_P_July; 3—Bit_P_June; 4—Bit_P_May; 5—Bit_P_September; 6—Bot_G_August; 7—Bot_G_July; 8—Bot_G_June; 9—Bot_G_May; 10—Bot_G_September; 11—Fili_P_August; 12—Fili_P_July; 13—Fili_P_June; 14—Fili_P_May; 15—Fili_P_September; 16—Suv_P_August; 17—Suv_P_July; 18—Suv_P_June; 19—Suv_P_May; 20—Suv_P_September. Total Cronbach’s ɑ = 0.970; explained variance = 55.773%. (**b**) Species distribution by month considering collection year: numbers of objects on the biplot: 1—May_2021; 2—June_2021; 3—July_2021; 4—August_2021; 5—September_2021; 6—May_2022; 7—June_2022; 8—July_2022; 9—August_2022; 10—September_2022; 11—May_2023; 12—June_2023; 13—July_2023; 14—August_2023; 15—September_2023. Total Cronbach’s ɑ = 0.981; explained variance = 67.018%. (**c**) Species distribution by habitat considering collection year: numbers of objects on the biplot: 1—Bit_P_2021; 2—Bit_P_2022; 3—Bit_P_2023; 4—Bot_G_2021; 5—Bot_G_2022; 6—Bot_G_2023; 7—Fili_P_2021; 8—Fili_P_2022; 9—Fili_P_2023; 10—Suv_P_2021; 11—Suv_P_2022; 12—Suv_P_2023. Total Cronbach’s ɑ = 0.983; explained variance = 68.823%.

The reproducibility of seasonal fluctuations in the abundance of most species and the significance of the factors “Site” and “Month” indicate the specificity of species distribution across sampling sites and accounting months. Significant differences in the diversity indices of drosophilid communities between parks, where the biotope is represented by mixed and predominantly small-leaved species or broad-leaved species, also support the specificity of species distribution. However, similar diversity indices can be obtained from very different sets of species, and conversely, the same set of species, with changes in the abundance of some of them, can yield significant differences in the diversity indices. To verify the nature of species distribution depending on the sampling site, month, and year, a Categorical Principal Components Analysis (CATPCA) was conducted using species abundance data of the samples as the analyzed variables and the pairwise combinations of “Site”, “Month”, and “Year” as the variable labels. The results of the analysis are presented in Figure 9. All three model variants describing species distribution under the considered conditions have high Cronbach’s α values (Figure 9, figure captions), confirming the reliability of the obtained results.

The species distribution considering the variability by factors “Site” and “Month” (Figure 9a) shows the formation of three distinct clusters, representing the separation of species based on ecological and seasonal indicators. The species *D. testacea*, *C. fuscimana*, and *H. confusa* are primarily associated with spring samples, regardless of the collection site. In this case, their less frequent occurrence in autumn samples is not significant. The species *H. trivittata*, *D. immigrans*, *D. busckii*, *D. repleta*, *D. funebris*, *C. amoena*, *A. albilablis*, *L. maculata*, *L. quinquemaculata*, *M. poecilogastra*, and *D. subobscura* are mainly present in samples from Suvorovsky Park, irrespective of the summer and autumn months, and in July samples from Fili Park. Among them, some species are typical synanthropic species, while others are species whose larvae develop on polypore fungi. Although the remaining species form a general cloud, we can highlight *S. pallida*, *S. graminum*, *D. kuntzei*, *G. distigma*, and *D. bifasciata*, which occur in the summer and autumn in the biotopes of Bitsa Park and the Main Botanical Garden. The remaining non-specialized species are encountered with a similar probability in the summer and autumn periods across all four biotopes.

The species distribution considering the variability of the factors “Year” and “Month” is represented by four clusters. One of these clusters again includes the species *D. testacea*, *C. fuscimana*, and *H. confusa*. In this case, they are grouped based on their occurrence in the samples from September 2022 and May–June 2023. This suggests that the specific conditions of autumn 2022 facilitated better winter survival for these species and their higher prevalence in spring 2023. Interestingly, a similar pattern is observed for the conditions of September 2021 and May–June 2022, differing only in a shift along the y-axis (PC2) for the remaining months of 2021. For autumn 2021 and spring 2022, the highest occurrence compared to other periods is observed for *L. quinquemaculata*, *S. pallida*, and *D. busckii*, and with some probability, during this period and the summer months of 2021, for the species *D. repleta*, *D. funebris*, *D. immigrans*, *A. albilabris*, *C. amoena*, *D. bifasciata*, and *S. graminum*. The cluster of numerous species including *S. rufifrons*, *P. semivirgo*, *D. subsilvestris*, *D. melanogaster*, *D. obscura*, *D. transversa*, *M. poecilogastra*, *D. histrio*, *D. kuntzei*, and *D. phalerata* is shifted towards the summer months of 2022 and 2023, indicating the peak presence of these species in the corresponding samples. Finally, *L. maculata* and *H. trivittata* are strictly clustered around the values of PC1 and PC2, characterizing June, July, and August 2022 as well as July and September 2023. These months exhibited the highest positive deviations in the mean monthly temperatures, except for July 2023, where a significant increase in daytime temperatures was observed in the first and last weeks of the month. It can be inferred that such conditions were optimal for these species.

The species distribution considering the factors of year and sampling site showed the correspondence of species clusters to sites, regardless of the collection year (Figure 9c). This confirms the earlier conclusion that the factor “Year” does not significantly influence species abundance indicators when evaluated based on aggregated data. This clustering largely reproduces the species clustering pattern considering the month and sampling site (Figure 9a), grouping species typical for Suvorovsky Park (*H. trivittata*, *D. immigrans*, *D. busckii*, *D. repleta*, *D. funebris*, *C. amoena*, *A. albilabris*, *L. maculata*, *M. poecilogastra*, and *D. subobscura*). Species characteristic of Fili Park and Bitsevsky Park are represented in two broad clusters, generally corresponding to those described for the distribution considering the factors “Site” and “Month”. It is noteworthy that the Main Botanical Garden did not form a distinct cluster of its own species, with only *D. bifasciata* shifted along the PC1 axis towards values typical for this site. The Main Botanical Garden has an impoverished drosophilid community, with significantly reduced numbers of synanthropic species (*D. busckii*, *D. funebris*, *D. hudei*, *D. immigrans*, *D. melanogaster*, and *D. repleta*), species whose life cycles are associated with polypores and wood fungi (*L. maculata*, *L. quinquemaculata*, *D. histrio*, *H. confusa*, *H. trivittata*, and *M. poecilogastra*), and those inhabiting tree bark, wounds, and decaying wood (*A. albilabris*, *Ph. semivirgo*, *Ch. amoena*, *Ch. fuscimana*, *D. subobscura*, *D. subsilvestris*, and *S. rufifrons*). The low abundance of synanthropic species can be attributed to the unique location of the Main Botanical Garden, which is significantly buffered by surrounding parks, recreational areas, museums, and institutes. The reduced numbers of other species might be linked to the absence of old and decaying trees and the active maintenance practices of the Botanical Garden staff in caring for the tree species.

## 4. Discussion

Until recently, the Drosophilidae fauna of Moscow was poorly studied, with records of only 13 species in 5 genera [36,47,48]. However, our study has significantly expanded this knowledge by identifying 21 new species in 9 genera (Table 1). Presently, the complete faunal list of Drosophilidae in Moscow comprises 34 species across 11 genera. It is noteworthy that *Scaptomyza consimilis* Hackman, although previously recorded [47], was absent in our collection.

Our investigation involved the collection of 26,420 individuals, representing 33 species and 11 genera of Drosophilidae across four sites within four natural parks of Moscow, spanning from May to September. This study represents the first exploration of seasonal dynamics in fruit flies’ abundance and species diversity within the natural parks of a Russian city.

The lowest abundance of drosophilids was recorded in May (Figure 1). This is the only similarity in the dynamics of fly abundance when comparing collections from different years, aggregated across all sampling sites; the rest of the data on drosophilid abundance noticeably differ from year to year. In 2021, we observed an increase in drosophilid abundance in June, followed by a slight further increase in July, a decrease in August, and a sharp rise to the annual peak abundance in September (1812, 1829, 1478, and 2125 individuals from June to September, respectively). In 2022, we observed two peaks in drosophilid abundance in June and September, with a decrease in July and August (2490, 2121, 1507, and 2518 individuals from June to September, respectively). Finally, in 2023, there was an increase in drosophilid abundance in June, reaching a plateau in July, followed by a very gradual decrease in August and September (1897, 1905, 1801, and 1780 individuals from June to September, respectively).

It is likely that the minimal abundance of drosophilids in May is associated with them being in puparia during and shortly after wintering and the gradual emergence of adults during this period. The difference in seasonal dynamics in other months may be related to the climatic characteristics of each year of the study.

The application of a non-parametric Kruskal–Wallis ANOVA by rank and the median test show no influence of the “Year” factor on species abundance. These results contradict the earlier conclusion regarding differences in the seasonal dynamics of communities from 2021 to 2023 based on aggregated data across all sites. This contradiction can be explained by the influence of the factors “Site” and “Month” on species abundance, which significantly increases the variance, masking differences in the analyzed samples. One could argue that in the CATPCA considering site and year of collection (Figure 9c), the weights of PCA1 and PCA2 are mainly determined by site diversity, also suggesting a minimal influence of the “Year” factor. However, this result is reproduced with proportional changes in species abundance across months, even considering the specific climatic features of the year. The interaction of these factors in species clustering is evident from the CATPCA results considering the month and year of collection (Figure 9b).

Seasonal changes in the temperature and precipitation intensity affect the biotopes of Moscow parks differently. A detailed analysis of the dynamics of ecological indicators, including diversity indices (Shannon–Wiener), the Simpson’s dominance index, and equitability indices, revealed a consistent pairwise similarity of sites with similar tree compositions over all three years. The non-randomness of this similarity is confirmed by the results of variance analysis and principal component analysis. Bitsevsky Park and Fili Park are predominantly represented by mixed forests, with a significant prevalence of deciduous species such as birch, aspen, alder, and the like. The diversity indices of these two parks in 2022 reached maximum values against the backdrop of record-high temperatures from July to August and a lack of rainfall in June and August. Presumably, the favorable microclimate for the drosophilid community in these locations is associated with the elevated temperature. The species composition of these communities is similar, as indicated by the results of the CATPCA considering the site and month of collection (Figure 9a) and the CATPCA considering the site and year of collection (Figure 9c). The differences are primarily in the quantitative assessments of species abundance.

The majority of the territory of the Main Botanical Garden and Suvorovsky Park is occupied by predominantly broad-leaved trees such as oak, maple, ash, and others. The characteristic maximum diversity index values for these sites in 2023 were observed against the backdrop of decreased temperatures in May, June, and July, but with twice the amount of rainfall in July and near normal or close to normal values in June and August. It is possible that humidity has the greatest influence on creating a favorable microclimate for drosophilids in these locations during the summer months. It is important to note the significant differences in the species composition and abundance of the drosophilid communities, which do not hinder the similar dynamics of seasonal changes in diversity indices. This similarity is evidently due to the proportional changes in species abundance in response to the influence of temperature and precipitation in habitats with similar tree species composition.

Our results demonstrated that the dynamics of diversity indices are dependent on the temperature and precipitation of the month preceding the collection of imagos. This suggests that the earlier developmental stages, specifically larval populations, are sensitive to temperature and humidity. It is also interesting to note the dependence of the abundance of certain species in the spring, after overwintering, on their abundance in August and September, before entering hibernation. Currently, it is difficult to assert that this influence is solely dependent on the temperature and precipitation in the autumn period, but with further observations, we hope to obtain more definitive confirmations and a more comprehensive list of species exhibiting such dependence.

To date, only two studies have examined the seasonal dynamics of drosophilids in Russia, both within the Mordovia State Nature Reserve. One study encompassed five distinct forest biotopes from early May to mid-October [49], while the other, focused primarily on vertical drosophilid distribution, surveyed similar deciduous forest biotopes from early June to mid-September [50]. Unfortunately, we cannot make direct comparisons between our data and the data from the reserve, as the drosophilid collections in the reserve were conducted only within one year each time and differed by the high positioning of the traps on tree trunks.

Our study revealed that the most abundant species in Moscow parks were *D. obscura* (11,103 individuals in three years), followed by *D. testacea* (3903 individuals) and *D. phalerata* (3680 specimens in three years). Interestingly, *D. obscura* was also identified as the most abundant species in both studies on seasonal drosophilid dynamics in the Mordovia Nature Reserve. Therefore, based on our three-year investigation of seasonal dynamics in parks and the reserve, *D. obscura* appears to be the most prevalent species in the European part of Russia.

Among our collection, we identified six synanthropic Drosophila species (*D. busckii*, *D. funebris*, *D. hydei*, *D. immigrans*, *D. melanogaster*, and *D. repleta*), well adapted to human environments, often reproducing in abundance in synanthropic conditions but typically occurring in small numbers in wild habitats. However, in Moscow’s natural parks, four synanthropic species (*D. busckii*, *D. immigrans*, *D. melanogaster*, and *D. repleta*) were captured in quantities ranging from 228 to 1768 individuals (Table 2). This can be attributed to people bringing various food items, fruits, and juices to the parks during certain seasons of the year, filling garbage bins with them and losing them under trees, which attracts synanthropic Drosophila and serves as breeding sites for them.

Recent studies have investigated the seasonal fluctuations and species diversity of drosophilids in semi-natural biotopes such as vineyards [51] and fruit orchards [52,53]. Surprisingly, the drosophilid species richness was significantly higher in Moscow natural parks (33 species) compared to semi-natural biotopes in France and Turkey, which are located in a warmer climate (17, 11, and 13 species, respectively).

Our study, conducted in only two Moscow parks over one year, served as a pilot experiment. Future research could expand to include more sites and parks in Moscow, as well as track fluctuations in drosophilid populations over multiple years.

In conclusion, our findings suggest that Moscow’s natural parks resemble forest biotopes more than semi-cultural environments such as gardens and vineyards or areas with significant anthropogenic influence like food markets or garbage dumps. Despite the urban setting, these parks support a diverse array of drosophilid species, indicating the high adaptability of “wild” drosophilids to exist within such environments. Unlike butterflies or beetles, these tiny fruit flies often go unnoticed by people as they quietly find suitable niches for feeding and reproduction, typically in tree wounds and fungi. With their small size and ability to occupy various ecological niches, drosophilids seem to thrive within urban parks, effectively transforming them into “islands of wildlife”. Further research into the ecological dynamics of urban parks and their inhabitants could provide valuable insights into the coexistence of wildlife and human populations in urban environments.

## 5. Conclusions

In this study, we provided the first comprehensive three-year exploration of seasonal fluctuations in the abundance and species diversity of drosophilids within Moscow’s natural parks. Our findings revealed a total of 26,420 drosophilid individuals spanning 11 genera and 33 species, with 21 species representing new additions to Moscow’s fauna. Among these species, *Drosophila obscura* Fll., *D. phalerata* Mg., and *D. testacea* Roser emerged as the most frequently captured in our traps. The peaks in drosophilid abundance varied between years, but the abundance was consistently lowest in May. The highest number of flies was collected in 2022 (9604 individuals), with slightly less in 2023 (8496 individuals) and even fewer in 2021 (8320 individuals). Additionally, 2022 also recorded the highest species diversity of drosophilids—33 species—while in 2021 and 2022, 28 species were found. Interestingly, August 2022 was the hottest and driest during these three years of collections, which could have influenced the increase in the number of fruit flies towards the end of summer and the beginning of autumn. When analyzing the population dynamics of individual species, the impact of the year of study is obscured by a high variance, which is associated with the effects of the “Month” and “Site” factors. The diversity indices exhibit a similar pattern among drosophilid communities inhabiting similar biotopes. The influence of specific climatic factors, such as the temperature and precipitation, affects species abundance and community diversity indices through the preimaginal developmental stages of drosophila. For several species, the population dynamics in the spring months, after emerging from overwintering, depend on the conditions preceding the winter period.

## Figures and Tables

**Table 1:** The faunal list of drosophilid flies in our collection.

Subfamily Steganinae	Subfamily Drosophilinae
1. * *Amiota albilabris* Roth	1. * *Chymomyza amoena* Lw.
2. * *Amiota alboguttata* Wahlberg	2. * *Chymomyza caudatula* Oldenberg
3. * *Amiota subtusradiata* Duda	3. * *Chymomyza costata* Ztt.
4. * *Gitona distigma* Mg.	4. * *Chymomyza fuscimana* Ztt.
5. * *Leucophenga maculata* Dufour	5. *Drosophila bifasciata* Pomini
6. * *Leucophenga quinquemaculata* Strobl	6. * *Drosophila busckii* Coq.
7. * *Phortica semivirgo* Maca	7. * *Drosophila funebris* F.
8. * *Stegana coleoptrata* Scop.	8. * *Drosophila histrio* Mg.
	9. * *Drosophila hydei* Sturtevant
	10. *Drosophila immigrans* Sturtevant
	11. * *Drosophila kuntzei* Duda
	12. * *Drosophila melanogaster* Mg.
	13. *Drosophila obscura* Fll.
	14. *Drosophila phalerata* Mg.
	15. *Drosophila repleta* Wollaston
	16. * *Drosophila subobscura* Collin
	17. *Drosophila subsilvestris* Hardy et Kaneshiro
	18. *Drosophila testacea* Roser
	19. *Drosophila transversa* Fll.
	20. * *Hirtodrosophila confusa* Staeger
	21. *Hirtodrosophila trivittata* Strobl
	22. *Mycodrosophila poecilogastra* Lw.
	23. *Scaptodrosophila rufifrons* Lw.
	24. *Scaptomyza pallida* Ztt.
	25. * *Scaptomyza graminum* Fll.

*—new species for the fauna of Moscow city.

**Table 2:** Number of Drosophilids collected in Moscow parks, 2021–2023.

Species	2021	2022	2023	Total
*Amiota albilabris*	0	5	4	9
*Amiota alboguttata*	0	3	0	3 *
*Amiota subtusradiata*	0	2	0	2 *
*Gitona distigma*	2	9	5	16
*Leucophenga maculata*	1	6	1	8
*Leucophenga quinquemaculata*	84	112	75	271
*Phortica semivirgo*	86	135	103	324
*Stegana coleoptrata*	0	1	0	1 *
*Chymomyza amoena*	5	18	4	27
*Chymomyza caudatula*	0	4	0	4 *
*Chymomyza costata*	0	1	0	1 *
*Chymomyza fuscimana*	9	7	7	23
*Drosophila bifasciata*	12	9	11	32
*Drosophila busckii*	90	93	45	228
*Drosophila funebris*	21	24	10	55
*Drosophila histrio*	233	375	213	821
*Drosophila hydei*	2	6	0	8
*Drosophila immigrans*	98	123	88	309
*Drosophila kuntzei*	178	319	122	619
*Drosophila melanogaster*	427	459	882	1768
*Drosophila obscura*	3515	3946	3642	11,103
*Drosophila phalerata*	1071	1587	1022	3680
*Drosophila repleta*	433	263	90	786
*Drosophila subobscura*	3	13	12	28
*Drosophila subsilvestris*	140	175	156	471
*Drosophila testacea*	1401	1122	1380	3903
*Drosophila transversa*	153	268	131	552
*Hirtodrosophila confusa*	19	23	38	80
*Hirtodrosophila trivittata*	11	22	8	41
*Mycodrosophila poecilogastra*	67	74	60	201
*Scaptodrosophila rufifrons*	238	372	330	940
*Scaptomyza graminum*	3	4	9	16
*Scaptomyza pallida*	18	24	48	90
Total	8320	9604	8496	26,420

*—species with a total number of less than 8 specimens were excluded from the analysis of the impacts of the year of collection, season, and point of collection on the abundance of the species.

**Table 3:** Average monthly temperature and humidity values in Moscow from May to September 2021–2023.

Year	Conditions	May (*)	June (*)	July (*)	August (*)	September (*)
2021	Temperature (°C)	14.0 (+0.4)	20.5 (+3.9)	22.2 (+2.5)	18.2 (+0.6)	10.9 (−1.0)
	Precipitation (mm)	94 (184%)	75 (97.4%)	42 (50%)	108 (140%)	69 (104%)
2022	Temperature (°C)	10.7 (−2.4)	19.5 (+2.2)	22.2 (+2.5)	23.1 (+5.5)	11.0 (−0.9)
	Precipitation (mm)	67 (131%)	35 (45.4%)	84 (100%)	4 (5.1%)	68 (103%)
2023	Temperature (°C)	12.7 (−0.9)	16.8 (−0.5)	18.5 (−1.2)	19.6 (+2.0)	15.0 (+3.1)
	Precipitation (mm)	38 (62%)	74 (100%)	160 (190%)	67 (86%)	8 (12%)

(*)—in parentheses, the deviation from the average monthly temperature and the percentage of the monthly precipitation norm are indicated.

**Table 4:** Wilks’s λ tests of the significance of differences in group means, taking into account locality, season, and fluctuations in the average monthly temperatures and precipitation: nested design.

Effect	Month^T^	λ	F	Effect df	Eror df	*p*
Site	1	0.0002	45.3	12	24.1	0.0000
Month (Site *)	1	0.0000	11.7	64	37.51	0.0000
Temperature (Site *)	1	0.0006	4.4	48	6.71	0.0000
Precipitation (Site *)	1	0.0002	4.5	64	37.51	0.0000
Site	2	0.1052	7.6	6	22	0.0002
Month (Site *)	2	0.0305	3.2	32	22	0.0027
Temperature (Site *)	2	0.5600	0.31	24	22	0.9969
Precipitation (Site *)	2	0.5397	0.2	32	22	0.9998
Site (Month *)	1	0.0202	12.06	30	60	0.0000
Month	1	0.0113	63.053	8	60	0.0000
Temperature (Month *)	1	0.7974	3.812	2	30	0.0335
Precipitation (Month *)	1	0.6041	4.299	4	60	0.0040
Site (Month *)	2	0.0420	37.51	6	58	0.0000
Month	2	0.0831	5.967	24	58	0.0000
Temperature (Month *)	2	0.4701	6.647	4	58	0.0002
Precipitation (Month *)	2	0.9062	0.732	4	58	0.5739

Over-parameterized model Type III decomposition. The main factor is indicated in parentheses. Month^T^—mean monthly temperature of the month preceding (1) or corresponding to (2) the time of sampling. Site *, Month *—the main factor in the given nested design. Month^T^ 1, 2—variants of using monthly averages of temperature and precipitation deviations from norms, obtained in the month before the collection or in the month of the collection, respectively.

**Table 5:** Values of constancy for four collection sites over the entire study period.

Species	Suvorovsky Park	Fili Park	The Main Botanical Garden	Bitsevsky Park
*D. obscura*	1	1	1	1
*D. phalerata*	2	2	2	2
*D. subsilvestris*	2	2	3	2
*D. testacea*	2	2	3	1
*S. rufifrons*	2	2	3	2
*L. quinquemaculata*	2	2	4	2
*D. histrio*	2	2	4	2
*M. poecilogastra*	2	2	6	6
*D. transversa*	3	2	2	2
*D. melanogaster*	3	2	3	3
*H. confusa*	3	3	4	4
*P. semivirgo*	3	3	5	2
*D. immigrans*	3	3	6	5
*D. funebris*	3	4	4	5
*H. trivittata*	3	4	6	4
*D. busckii*	4	3	6	6
*D. repleta*	4	3	6	6
*C. amoena*	4	4	4	6
*D. bifasciata*	4	4	4	4
*D. kuntzei*	4	4	4	2
*C. fuscimana*	4	4	5	4
*D. subobscura*	4	4	6	5
*G. distigma*	4	5	5	4
*A. albilabris*	4	5	6	6
*L. maculata*	4	5	6	6
*D. hydei*	4	5	6	6
*S. pallida*	5	5	4	3
*A. alboguttata*	5	5	6	6
*A. subtusradiata*	5	5	6	6
*C. caudatula*	5	5	6	5
*S. coleoptrata*	5	6	6	6
*C. costata*	5	6	6	6
*S. graminum*	6	6	5	4

**Table 6:** Influence of the site, year, and month Factors on species abundance (data combined for 3 years of study).

Species	Site					Year					Month				
	K-W Test		Median Test			K-W Test		Median Test			K-W Test		Median Test		
	H	*p*	χ2	df	*p*	H	*p*	χ2	df	*p*	H	*p*	χ2	df	*p*
*A. albilabris*	8.30	0.0403	8.15	3	0.0430	3.27	0.1948	3.33	2	0.1889	9.81	0.0437	10.00	4	0.0404
*G. distigma*	5.44	0.1421	5.79	3	0.1221	5.79	0.0552	6.09	2	0.0476	11.82	0.0187	11.39	4	0.0225
*L. maculata*	12.46	0.0060	12.59	3	0.0056	3.38	0.1843	3.33	2	0.1889	6.31	0.1774	6.30	4	0.1781
*L. quinquemaculata*	24.53	0.0000	20.36	3	0.0001	2.50	0.2867	3.39	2	0.1833	22.03	0.0002	15.20	4	0.0043
*P. semivirgo*	12.25	0.0066	7.87	3	0.0487	0.35	0.8392	0.54	2	0.7622	35.38	0.0000	30.81	4	0.0000
*C. amoena*	8.67	0.0341	8.86	3	0.0312	4.17	0.1240	2.73	2	0.2557	15.24	0.0042	16.53	4	0.0024
*C. fuscimana*	2.96	0.3974	2.86	3	0.4142	0.40	0.8199	0.48	2	0.7881	38.40	0.0000	39.37	4	0.0000
*D. bifasciata*	1.72	0.6331	1.39	3	0.7074	0.08	0.9607	0.00	2	1	40.36	0.0000	42.05	4	0.0000
*D. busckii*	22.43	0.0001	21.60	3	0.0001	0.62	0.7352	0.53	2	0.7659	11.62	0.0204	11.56	4	0.0210
*D. funebris*	10.42	0.0153	9.50	3	0.0233	1.34	0.5128	0.95	2	0.6218	22.44	0.0002	21.31	4	0.0003
*D. histrio*	19.88	0.0002	20.53	3	0.0001	0.28	0.8678	0.40	2	0.8187	29.55	0.0000	24.00	4	0.0001
*D. hydei*	9.38	0.0246	9.38	3	0.0246	3.09	0.2134	3.05	2	0.2171	2.01	0.7338	2.18	4	0.7024
*D. immigrans*	17.53	0.0006	16.20	3	0.0010	0.35	0.8398	0.60	2	0.7408	19.40	0.0007	20.25	4	0.0004
*D. kuntzei*	25.49	0.0000	15.48	3	0.0015	0.99	0.6081	0.95	2	0.6218	14.77	0.0052	18.60	4	0.0009
*D. melanogaster*	5.62	0.1319	3.21	3	0.3598	1.82	0.4023	1.74	2	0.4187	41.96	0.0000	37.90	4	0.0000
*D. obscura*	5.58	0.1337	4.53	3	0.2093	0.38	0.8257	0.40	2	0.8187	49.13	0.0000	42.67	4	0.0000
*D. phalerata*	19.13	0.0003	23.73	3	0.0000	0.00	0.9980	1.20	2	0.5488	32.01	0.0000	18.00	4	0.0012
*D. repleta*	20.54	0.0001	20.18	3	0.0002	0.51	0.7765	0.53	2	0.7659	14.76	0.0052	16.00	4	0.0030
*D. subobscura*	10.12	0.0176	10.23	3	0.0167	2.76	0.2519	2.22	2	0.3302	15.89	0.0032	16.53	4	0.0024
*D. subsilvestris*	8.29	0.0404	13.39	3	0.0039	0.31	0.8569	0.13	2	0.9352	43.50	0.0000	29.87	4	0.0000
*D. testacea*	5.36	0.1472	2.40	3	0.4936	0.34	0.8449	0.00	2	1	50.96	0.0000	48.00	4	0.0000
*D. transversa*	6.71	0.0818	6.96	3	0.073	3.09	0.2137	2.14	2	0.3425	42.94	0.0000	28.53	4	0.0000
*H. confusa*	4.97	0.1739	3.97	3	0.2644	1.24	0.5377	1.21	2	0.5455	39.47	0.0000	37.44	4	0.0000
*H. trivittata*	11.86	0.0079	12.55	3	0.0057	1.84	0.3990	1.08	2	0.5832	26.07	0.0000	24.18	4	0.0001
*M. poecilogastra*	36.78	0.0000	40.00	3	0.0000	0.13	0.9352	0.00	2	1	9.99	0.0406	10.00	4	0.0404
*S. rufifrons*	9.50	0.0233	11.47	3	0.0095	2.13	0.3450	5.20	2	0.0743	39.50	0.0000	24.00	4	0.0001
*S. graminum*	13.00	0.0046	12.94	3	0.0048	0.92	0.6312	0.78	2	0.6756	15.56	0.0037	16.21	4	0.0028
*S. pallida*	9.92	0.0193	7.20	3	0.0658	2.12	0.3473	1.95	2	0.3772	23.09	0.0001	23.25	4	0.0001
B-H 0.05		0.0304			0.0286		--			--		0.0464			0.0464

K-W test—Kruskal-Wallis ANOVA by rank. Benjamini and Hochberg corrected values for species–site samples: for the Kruskal-Wallis ANOVA for the 0.05 level of significance, *p* < 0.0304; for the 0.01 level of significance, *p* < 0.0029; for the median test for the 0.05 level of significance, *p* < 0.0286; and for the 0.01 level of significance, *p* < 0.0029. Benjamini and Hochberg corrected values for species–month samples: for the Kruskal–Wallis ANOVA and the median test for the 0.05 level of significance critical value, *p* < 0.0464; and for the 0.01 level of significance critical value, *p* < 0.0079. For species–year samples, Benjamini and Hochberg correction did not show significant values.

**Table 7:** Dependence of species abundance on the month of sampling for each year of the study.

Species	2021		2022		2023	
	K-W p	Med. P	K-W p	Med. P	K-W p	Med. P
*A. albilabris*	1	1	0.2186	0.1796	0.1903	0.1796
*G. distigma*	0.5303	0.5037	**0.0098**	**0.0081**	0.2317	0.1796
*L. maculata*	0.406	0.3783	0.3505	0.3576	0.406	0.3783
*L. quinquemaculata*	0.183	0.2215	0.0759	0.0916	0.1026	0.2472
*P. semivirgo*	**0.0093**	**0.0081**	0.0693	0.0916	**0.0062**	**0.0081**
*C. amoena*	0.1313	0.1117	0.2501	0.2472	0.1294	0.1117
*C. fuscimana*	**0.0029**	**0.0022**	0.0324	0.0306	**0.017**	**0.011**
*D. bifasciata*	**0.0046**	**0.0022**	0.0419	0.0386	**0.0033**	**0.0022**
*D. busckii*	0.4614	0.5037	0.2915	0.2548	0.1336	0.1117
*D. funebris*	0.0835	0.143	0.0912	0.0404	0.0426	0.0386
*D. histrio*	**0.018**	0.0916	0.0382	0.0916	0.0724	0.0916
*D. hydei*	0.5303	0.5037	0.689	0.6711	1	1
*D. immigrans*	0.259	0.2215	0.0673	0.0481	0.259	0.2215
*D. kuntzei*	0.0906	0.0276	0.1811	0.0916	0.5516	0.4754
*D. melanogaster*	**0.0022**	**0.002**	**0.0062**	**0.0073**	**0.0038**	**0.003**
*D. obscura*	**0.0024**	**0.003**	**0.0036**	**0.0073**	**0.0027**	**0.003**
*D. phalerata*	**0.0174**	0.0916	0.0884	0.1992	0.0197	0.1992
*D. repleta*	0.3393	0.2548	0.2601	0.2215	0.1336	0.1117
*D. subobscura*	0.2021	0.1796	0.1282	**0.0882**	0.1136	0.0949
*D. subsilvestris*	**0.0153**	0.0916	**0.0068**	0.0916	**0.0033**	**0.0116**
*D. testacea*	**0.0027**	**0.003**	**0.0022**	**0.003**	**0.0018**	**0.003**
*D. transversa*	**0.002**	**0.003**	**0.0083**	0.0404	**0.0046**	0.0404
*H. confusa*	0.0348	0.0386	**0.0114**	0.0073	**0.0029**	**0.003**
*H. trivittata*	**0.0183**	**0.012**	0.0579	0.1117	**0.0747**	**0.0916**
*M. poecilogastra*	0.4662	0.5037	0.5079	0.5037	0.519	0.5037
*S. rufifrons*	**0.0042**	0.0276	0.042	0.0916	**0.0044**	0.0404
*S. graminum*	0.5296	0.5037	0.2317	0.1796	0.1311	0.1117
*S. pallida*	0.3012	0.2548	0.0794	0.0882	0.0251	**0.0116**
B-H 0.05	0.0214	0.0143	0.0125	0.0089	0.0179	0.0161
B-H 0.01	--	--	--	--	--	--

K-W test—Kruskal-Wallis ANOVA by rank. B-H 0.05—Benjamini and Hochberg corrected values for the 0.05 level of significance. None of the multiple tests yielded significant results. Significant effects of the “Month” factor on species abundance are highlighted in bold.

## Data Availability

The data presented in the study are available in the article.

## Supplementary Materials

The following supporting information can be downloaded at: https://www.mdpi.com/article/10.3390/insects15060398/s1, Figure S1: A map of Moscow with the territories of the four parks highlighted. Figure S2: Seasonal dynamics of diversity indices (Shannon–Wiener) by sites. The x-axis represents the collection months, while the y-axis represents the index values. a—Site 1, Bitsevsky Forest Natural Historical Park; b—Site 2, Fili Park; c—Site 3, the Main Botanical Garden; d—Site 4, Suvorovsky Park.; Table S1: Descriptive statistics for samples pooled by Drosophila collection sites. Table S2: Descriptive statistics for samples pooled by Drosophila collection years. Table S3: Descriptive statistics for samples pooled by Drosophila collection month. Table S4: The influence of the “Year” factor on the abundance of species in collections from city parks. Table S5: The influence of the “Site” factor on the abundance of species in collections from city parks. Table S6: Influence of the “Year” factor on the abundance of species in monthly collections. Table S7.
