# Supplementary material for: Seasonal Dynamics of Fruit Flies (Diptera: Drosophilidae) in Natural Parks of Moscow City, Russia
Source: Insects. 2024 May 29;15(6):398. doi: 10.3390/insects15060398 (PMC11204148; doi:10.3390/insects15060398)
Supplement: Supplementary file 1 [file insects-15-00398-s001.zip › insects-2962517-supplementary-EE done.pdf]

Supplementary materials.

Figure S1. A map of Moscow with the territories of the four parks highlighted.

Typical plant biotopes of the four Moscow parks:

Bitsevsky Forest Natural Historical Park

https://yandex.ru/maps/org/bittsevskiy_les/235963440654/gallery/?ll=37.553563%2C55.607552&photos%5Bbusiness%5D=235963440654&photos%5Bid%5D=urn%3Ayandex%3Asprav%3Aphoto%3A4912283&tab=gallery&z=12.24

https://yandex.ru/maps/org/bittsevskiy_les/235963440654/gallery/?ll=37.553563%2C55.607552&photos%5Bbusiness%5D=235963440654&photos%5Bid%5D=urn%3Ayandex%3Asprav%3Aphoto%3A4912138&z=12

Fili Park

https://yandex.ru/maps/org/park_fili/1072958798/gallery/?ll=37.488091%2C55.745565&photos%5Bbusiness%5D=1072958798&photos%5Bid%5D=urn%3Ayandex%3Asprav%3Aphoto%3A191644679&tab=gallery&z=13.62

https://yandex.ru/maps/org/park_fili/1072958798/gallery/?ll=37.488091%2C55.745565&photos%5Bbusiness%5D=1072958798&photos%5Bid%5D=urn%3Ayandex%3Asprav%3Aphoto%3A4912866&tab=gallery&z=13.62

Suvorovsky Park

https://yandex.ru/maps/213/moscow/geo/suvorovskiy_park/121406152/?ll=37.438951%2C55.742293&photos%5Bid%5D=tuhx4ZFOlFP9-SyKjFFW8g&photos%5Buri%5D=ymapsbm1%3A%2F%2Fgeo%3Fdata%3DCgkxMjE0MDYxNTISO9Cg0L7RgdGB0LjRjywg0JzQvtGB0LrQstCwLCDQodGD0LLQvtGA0L7QstGB0LrQuNC5INC_0LDRgNC6IgoNTsIVQhXE915C&tab=gallery&z=14.73

https://yandex.ru/maps/213/moscow/geo/suvorovskiy_park/121406152/?ll=37.438951%2C55.742293&photos%5Bid%5D=O5MC7TwHm561D3aOqvXcWQ&photos%5Buri%5D=ymapsbm1%3A%2F%2Fgeo%3Fdata%3DCgkxMjE0MDYxNTISO9Cg0L7RgdGB0LjRjywg0JzQvtGB0LrQstCwLCDQodGD0LLQvtGA0L7QstGB0LrQuNC5INC_0LDRgNC6IgoNTsIVQhXE915C&tab=gallery&z=14.73

Main Botanical Garden.

https://yandex.ru/maps/213/moscow/geo/glavny_botanicheskiy_sad_imeni_n_v_tsitsina_rossiyskoy_akademii_nauk/121409112/?ll=37.604007%2C55.838470&panorama%5Bdirection%5D=159.023342%2C1.000000&panorama%5Bfull%5D=true&panorama%5Bpoint%5D=37.612910%2C55.843546&panorama%5Bspan%5D=123.258738%2C60.000000&z=14.56

https://yandex.ru/maps/org/botanicheskiy_sad_ran/21634514483/gallery/?display-text=%D0%B3%D0%BB%D0%B0%D0%B2%D0%BD%D1%8B%D0%B9%20%D0%B1%D0%BE%D1%82%D0%B0%D0%BD%D0%B8%D1%87%D0%B5%D1%81%D0%BA%D0%B8%D0%B9%20%D1%81%D0%B0%D0%B4%20%D0%B8%D0%BC%D0%B5%D0%BD%D0%B8%20%D0%BD%20%D0%B2%20%D1%86%D0%B8%D1%86%D0%B8%D0%BD%D0%B0%20%D1%80%D0%BE%D1%81%D1%81%D0%B8%D0%B9

%D1%81%D0%BA%D0%BE%D0%B9%20%D0%B0%D0%BA%D0%B0%D0%B4%D0%B5%D0%BC%D0%B8%D0%B8%20%D0%BD%D0%
B0%D1%83%D0%BA&ll=37.591720%2C55.789032&mode=search&photos%5Bbusiness%5D=21634514483&photos%5Bid%5D=urn%3Ayandex
%3Asprav%3Aphoto%3A13229990_2a0000018fb9834adef12a5eb553fc30e9dc&sll=37.438951%2C55.742293&sspn=0.047815%2C0.014800&tab=g
allery&text=%D0%B3%D0%BB%D0%B0%D0%B2%D0%BD%D1%8B%D0%B9%20%D0%B1%D0%BE%D1%82%D0%B0%D0%BD%D0%B8
%D1%87%D0%B5%D1%81%D0%BA%D0%B8%D0%B9%20%D1%81%D0%B0%D0%B4%20%D0%B8%D0%BC%D0%B5%D0%BD%D0%B8
%20%D0%BD%20%D0%B2%20%D1%86%D0%B8%D1%86%D0%B8%D0%BD%D0%B0%20%D1%80%D0%BE%D1%81%D1%81%D0%B8%
D0%B9%D1%81%D0%BA%D0%BE%D0%B9%20%D0%B0%D0%BA%D0%B0%D0%B4%D0%B5%D0%BC%D0%B8%D0%B8%20%D0%BD
%D0%B0%D1%83%D0%BA&z=11.73

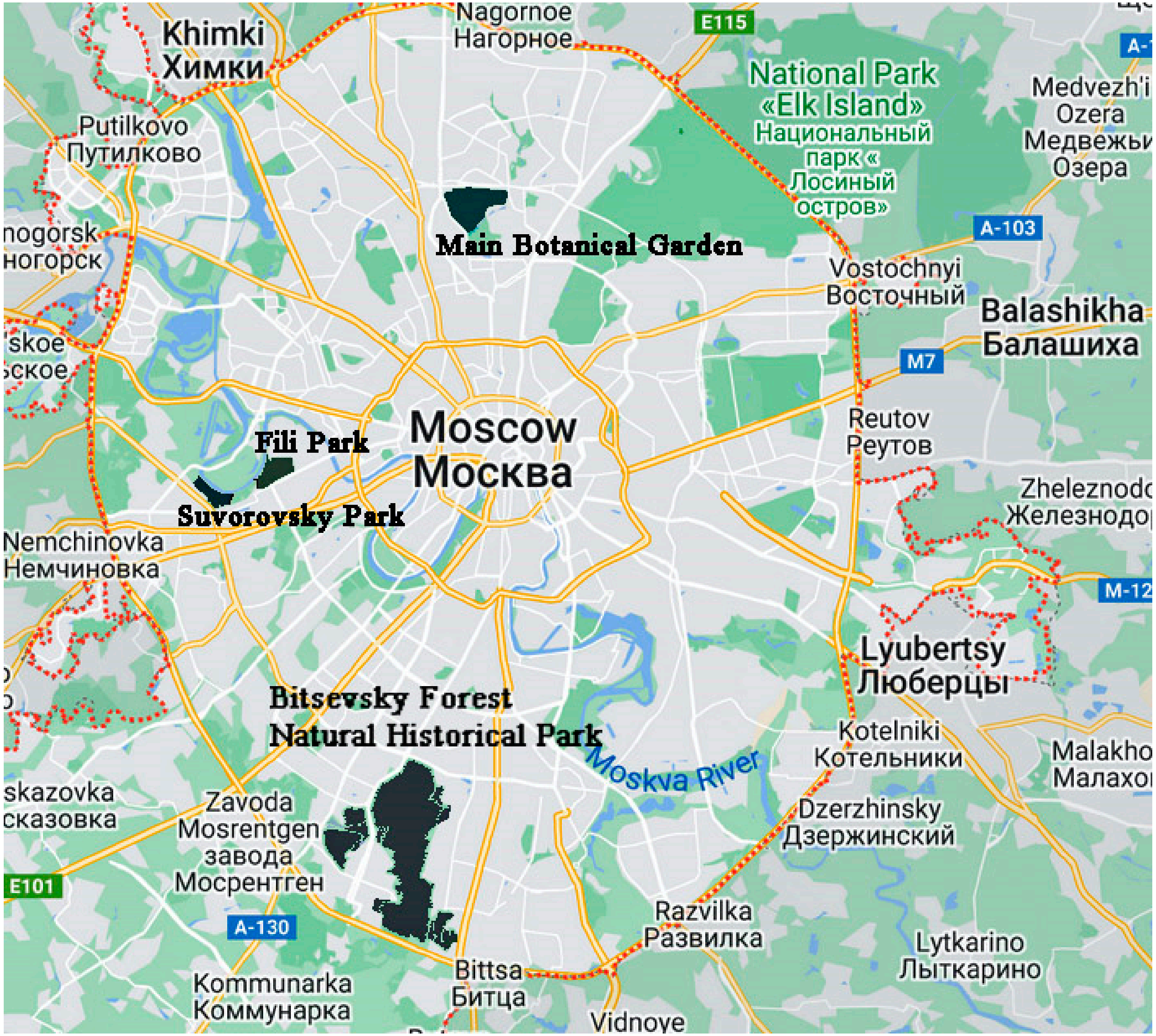

Khimki
Химки
Nagornoe
Нагорное
E115
National Park «Elk Island»
Национальный парк «Лосиный остров»
Medvezh'i Ozera
Медвежьи Озера
A-103
Putilkovo
Путилково
Main Botanical Garden
Vostochnyi
Восточный
Balashikha
Балашиха
M7
Moscow
Москва
Reutov
Реутов
Fili Park
Suvorovsky Park
Nemchinovka
Немчиновка
M-12
Lyubertsy
Люберцы
Bitsevsky Forest
Natural Historical Park
Moskva River
Kotelniki
Котельники
Zavoda Mosrentgen
завода Мосрентген
Dzerzhinsky
Дзержинский
E101
A-130
Razvilka
Развилка
Lytkarino
Лыткарино
Kommunarka
Коммунарка
Bittsa
Битца
Vidnoye

Figure S2. Seasonal dynamics of diversity indices (Shannon–Wiener) by sites. The x-axis represents the collection months, while the y-axis represents the index values. a—Site 1, Bitsevsky Forest Natural Historical Park; b—Site 2, Fili Park; c—Site 3, the Main Botanical Garden; d—Site 4, Suvorovsky Park.

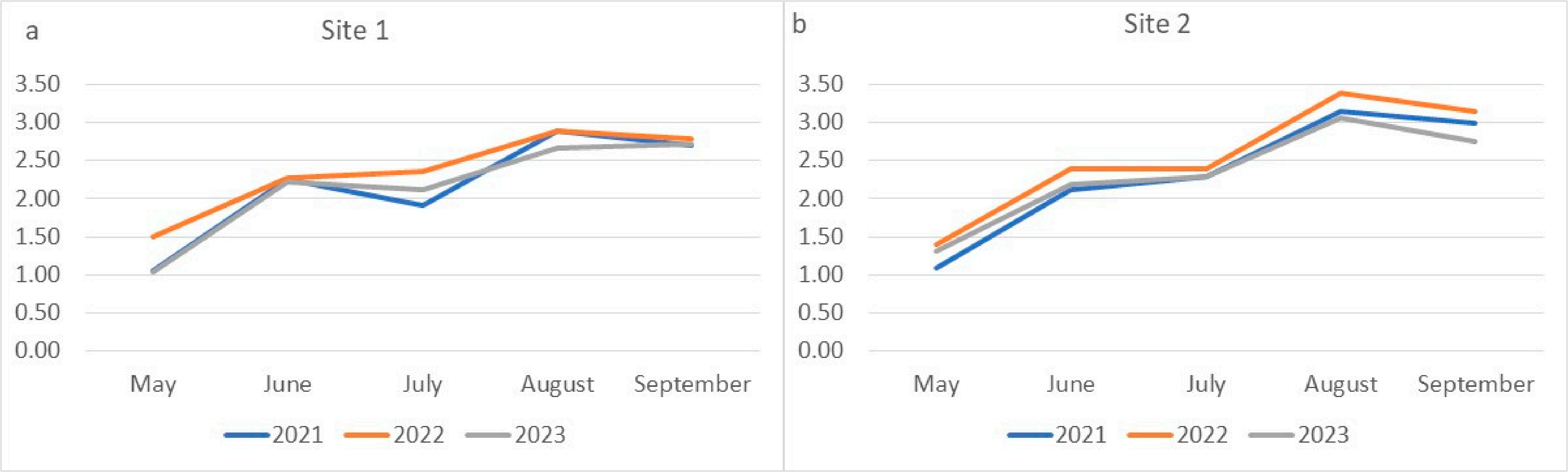

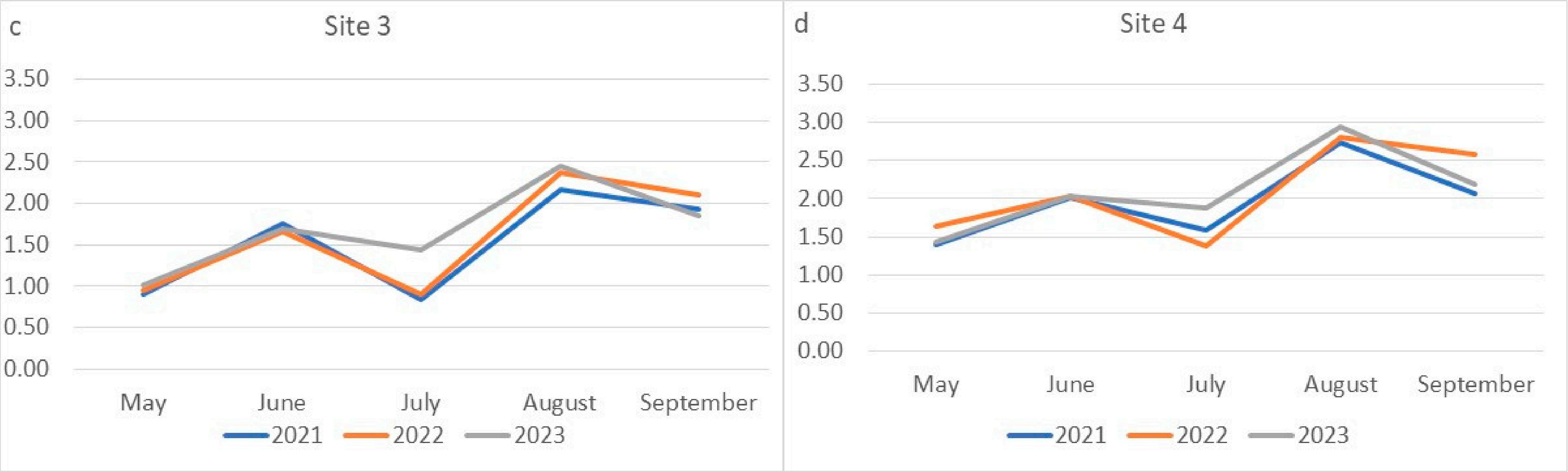

Table S1. Descriptive statistics for samples pooled by Drosophila collection sites.

Species	Site	Valid N	Mean	Median	Sum	Min	Max	Lower Q	Upper Q	Std. Dev.	Skewness	Std. Err.	Kurtosis	Std. Err.	K-S *d*	K-S *p*	Lilliefors *p*
A. albilabris	1	15	0.00	0	0	0	0	0	0	0.000			0.00				
A. albilabris	2	15	0.13	0	2	0	1	0	0	0.352	2.40	0.580	4.35	1.121	0.51430	<0.01	<0.01
A. albilabris	3	15	0.00	0	0	0	0	0	0	0.000			0.00				
A. albilabris	4	15	0.47	0	7	0	2	0	1	0.834	1.40	0.580	0.14	1.121	0.44548	<0.01	<0.01
G. distigma	1	15	0.53	0	8	0	3	0	1	0.834	2.01	0.580	4.87	1.121	0.33879	<0.05	<0.01
G. distigma	2	15	0.13	0	2	0	2	0	0	0.516	3.87	0.580	15.00	1.121	0.53521	<0.01	<0.01
G. distigma	3	15	0.13	0	2	0	1	0	0	0.352	2.40	0.580	4.35	1.121	0.51430	<0.01	<0.01
G. distigma	4	15	0.27	0	4	0	1	0	1	0.458	1.18	0.580	-0.73	1.121	0.45324	<0.01	<0.01
L. maculata	1	15	0.00	0	0	0	0	0	0	0.000			0.00				
L. maculata	2	15	0.07	0	1	0	1	0	0	0.258	3.87	0.580	15.00	1.121	0.53521	<0.01	<0.01
L. maculata	3	15	0.00	0	0	0	0	0	0	0.000			0.00				

L. maculata	4	15	0.47	0	7	0	3	0	1	0.834	2.25	0.580	5.78	1.121	0.37882	<0.05	<0.01
L. quinquemaculata	1	15	4.53	4	68	0	12	2	7	3.441	0.63	0.580	-0.04	1.121	0.16159	>0.20	>0.20
L. quinquemaculata	2	15	5.93	5	89	0	13	3	9	4.044	0.23	0.580	-1.08	1.121	0.15038	>0.20	>0.20
L. quinquemaculata	3	15	0.60	0	9	0	4	0	1	1.121	2.34	0.580	5.89	1.121	0.37039	<0.05	<0.01
L. quinquemaculata	4	15	7.00	7	105	0	14	5	10	3.928	-0.28	0.580	-0.41	1.121	0.11083	>0.20	>0.20
P. semivirgo	1	15	7.40	3	111	0	31	1	11	9.833	1.52	0.580	1.25	1.121	0.24956	>0.20	<0.05
P. semivirgo	2	15	7.40	2	111	0	27	0	14	9.249	0.98	0.580	-0.42	1.121	0.28287	<0.20	<0.01
P. semivirgo	3	15	0.47	0	7	0	3	0	0	1.060	2.18	0.580	3.45	1.121	0.47011	<0.01	<0.01
P. semivirgo	4	15	6.33	1	95	0	22	0	11	7.622	0.94	0.580	-0.42	1.121	0.29128	<0.15	<0.01
C. amoena	1	15	0.00	0	0	0	0	0	0	0.000			0.00				
C. amoena	2	15	0.73	0	11	0	3	0	1	0.961	1.17	0.580	0.59	1.121	0.31059	<0.10	<0.01
C. amoena	3	15	0.33	0	5	0	2	0	1	0.617	1.79	0.580	2.63	1.121	0.43876	<0.01	<0.01
C. amoena	4	15	0.73	0	11	0	5	0	1	1.387	2.40	0.580	6.31	1.121	0.36816	<0.05	<0.01
C. fuscimana	1	15	0.47	0	7	0	2	0	1	0.640	1.08	0.580	0.40	1.121	0.36707	<0.05	<0.01
C. fuscimana	2	15	0.47	0	7	0	2	0	1	0.743	1.33	0.580	0.47	1.121	0.40163	<0.05	<0.01
C. fuscimana	3	15	0.13	0	2	0	1	0	0	0.352	2.40	0.580	4.35	1.121	0.51430	<0.01	<0.01
C. fuscimana	4	15	0.47	0	7	0	2	0	1	0.743	1.33	0.580	0.47	1.121	0.40163	<0.05	<0.01
D. bifasciata	1	15	0.67	0	10	0	2	0	1	0.816	0.74	0.580	-1.02	1.121	0.32623	<0.10	<0.01
D. bifasciata	2	15	0.60	0	9	0	3	0	1	0.986	1.49	0.580	1.15	1.121	0.39532	<0.05	<0.01
D. bifasciata	3	15	0.60	0	9	0	2	0	2	0.910	0.97	0.580	-1.10	1.121	0.41177	<0.01	<0.01
D. bifasciata	4	15	0.27	0	4	0	1	0	1	0.458	1.18	0.580	-0.73	1.121	0.45324	<0.01	<0.01
D. busckii	1	15	0.00	0	0	0	0	0	0	0.000			0.00				
D. busckii	2	15	13.40	4	201	0	41	0	29	15.217	0.62	0.580	-1.29	1.121	0.26496	>0.20	<0.01
D. busckii	3	15	0.00	0	0	0	0	0	0	0.000			0.00				
D. busckii	4	15	1.80	0	27	0	12	0	2	3.406	2.33	0.580	5.49	1.121	0.32619	<0.10	<0.01
D. funebris	1	15	0.33	0	5	0	2	0	0	0.724	1.98	0.580	2.55	1.121	0.47745	<0.01	<0.01

D. funebris	2	15	0.47	0	7	0	3	0	1	0.834	2.25	0.580	5.78	1.121	0.37882	<0.05	<0.01
D. funebris	3	15	0.73	0	11	0	2	0	2	0.884	0.60	0.580	-1.49	1.121	0.33002	<0.10	<0.01
D. funebris	4	15	2.13	1	32	0	8	0	3	2.560	1.45	0.580	1.11	1.121	0.27103	<0.20	<0.01
D. histrio	1	15	30.00	18	450	0	124	5	44	35.707	1.72	0.580	2.62	1.121	0.22216	>0.20	<0.05
D. histrio	2	15	12.87	14	193	0	36	2	21	11.147	0.50	0.580	-0.50	1.121	0.14528	>0.20	>0.20
D. histrio	3	15	0.87	0	13	0	4	0	2	1.407	1.33	0.580	0.29	1.121	0.39764	<0.05	<0.01
D. histrio	4	15	11.00	13	165	0	25	1	20	9.266	0.06	0.580	-1.61	1.121	0.20604	>0.20	<0.01
D. hydei	1	15	0.00	0	0	0	0	0	0	0.000			0.00				
D. hydei	2	15	0.07	0	1	0	1	0	0	0.258	3.87	0.580	15.00	1.121	0.53521	<0.01	<0.01
D. hydei	3	15	0.00	0	0	0	0	0	0	0.000			0.00				
D. hydei	4	15	0.47	0	7	0	3	0	1	0.915	2.05	0.580	3.65	1.121	0.42822	<0.01	<0.01
D. immigrans	1	15	0.40	0	6	0	3	0	0	0.910	2.31	0.580	4.66	1.121	0.46983	<0.01	<0.01
D. immigrans	2	15	17.93	3	269	0	52	0	41	21.019	0.54	0.580	-1.59	1.121	0.29463	<0.15	<0.01
D. immigrans	3	15	0.00	0	0	0	0	0	0	0.000			0.00				
D. immigrans	4	15	2.27	2	34	0	6	0	4	2.154	0.20	0.580	-1.50	1.121	0.25371	>0.20	<0.05
D. kuntzei	1	15	39.27	36	589	0	121	5	76	38.250	0.79	0.580	-0.32	1.121	0.16203	>0.20	>0.20
D. kuntzei	2	15	0.53	0	8	0	5	0	1	1.302	3.28	0.580	11.49	1.121	0.39229	<0.05	<0.01
D. kuntzei	3	15	0.73	0	11	0	5	0	1	1.486	2.18	0.580	4.41	1.121	0.42245	<0.01	<0.01
D. kuntzei	4	15	0.73	0	11	0	5	0	1	1.387	2.40	0.580	6.31	1.121	0.36816	<0.05	<0.01
D. melanogaster	1	15	36.80	9	552	0	234	0	60	60.490	2.77	0.580	8.76	1.121	0.27147	<0.20	<0.01
D. melanogaster	2	15	42.60	7	639	0	215	2	54	61.454	1.98	0.580	3.89	1.121	0.25214	>0.20	<0.05
D. melanogaster	3	15	7.53	1	113	0	46	0	12	13.266	2.21	0.580	4.73	1.121	0.30906	<0.10	<0.01
D. melanogaster	4	15	30.93	8	464	0	131	0	48	43.590	1.43	0.580	0.78	1.121	0.25680	>0.20	<0.05
D. obscura	1	15	211.27	199	3169	100	380	111	280	100.982	0.51	0.580	-1.09	1.121	0.16808	>0.20	>0.20
D. obscura	2	15	185.60	202	2784	77	321	91	259	91.827	0.17	0.580	-1.54	1.121	0.23541	>0.20	<0.05
D. obscura	3	15	140.27	103	2104	45	319	60	203	93.989	0.82	0.580	-0.74	1.121	0.21006	>0.20	<0.10
D. obscura	4	15	203.07	213	3046	75	412	111	279	99.114	0.43	0.580	-0.40	1.121	0.16019	>0.20	>0.20
D. phalerata	1	15	134.07	118	2011	0	521	49	160	134.977	1.87	0.580	4.35	1.121	0.25034	>0.20	<0.05
D. phalerata	2	15	67.33	54	1010	0	137	30	109	48.115	-0.04	0.580	-1.40	1.121	0.18314	>0.20	<0.20
D. phalerata	3	15	36.60	34	549	0	92	10	64	29.691	0.39	0.580	-0.93	1.121	0.10884	>0.20	>0.20

D. phalerata	4	15	7.33	5	110	0	29	1	9	8.997	1.74	0.580	2.17	1.121	0.29224	<0.15	<0.01
D. repleta	1	15	0.00	0	0	0	0	0	0	0.000			0.00				
D. repleta	2	15	48.73	26	731	0	203	0	96	60.937	1.26	0.580	1.35	1.121	0.25473	>0.20	<0.05
D. repleta	3	15	0.00	0	0	0	0	0	0	0.000			0.00				
D. repleta	4	15	3.67	0	55	0	13	0	8	4.670	0.85	0.580	-0.83	1.121	0.31715	<0.10	<0.01
D. subobscura	1	15	0.33	0	5	0	3	0	0	0.816	2.89	0.580	8.87	1.121	0.45845	<0.01	<0.01
D. subobscura	2	15	0.53	0	8	0	2	0	1	0.743	1.07	0.580	-0.11	1.121	0.36350	<0.05	<0.01
D. subobscura	3	15	0.00	0	0	0	0	0	0	0.000			0.00				
D. subobscura	4	15	1.00	0	15	0	5	0	2	1.464	1.73	0.580	2.98	1.121	0.28607	<0.15	<0.01
D. subsilvestris	1	15	11.87	8	178	0	33	2	22	11.544	0.67	0.580	-0.97	1.121	0.19068	>0.20	<0.15
D. subsilvestris	2	15	9.40	5	141	0	28	1	15	9.977	0.76	0.580	-0.82	1.121	0.20373	>0.20	<0.10
D. subsilvestris	3	15	2.00	2	30	0	12	0	3	3.024	2.84	0.580	9.51	1.121	0.30376	<0.15	<0.01
D. subsilvestris	4	15	8.13	7	122	0	23	1	12	8.476	0.83	0.580	-0.66	1.121	0.19429	>0.20	<0.15
D. testacea	1	15	92.20	90	1383	1	308	2	121	97.915	1.01	0.580	0.30	1.121	0.21067	>0.20	<0.10
D. testacea	2	15	70.07	74	1051	0	208	1	118	68.115	0.55	0.580	-0.60	1.121	0.23396	>0.20	<0.05
D. testacea	3	15	50.67	58	760	0	144	0	78	48.913	0.42	0.580	-0.86	1.121	0.24504	>0.20	<0.05
D. testacea	4	15	47.27	43	709	0	115	1	85	44.506	0.20	0.580	-1.59	1.121	0.25073	>0.20	<0.05
D. transversa	1	15	13.53	14	203	0	41	6	18	11.269	1.05	0.580	1.26	1.121	0.14591	>0.20	<0.05
D. transversa	2	15	10.40	10	156	0	34	4	15	8.895	1.26	0.580	2.45	1.121	0.13978	>0.20	>0.20
D. transversa	3	15	6.87	5	103	0	28	1	11	7.386	1.80	0.580	4.06	1.121	0.22613	>0.20	<0.05
D. transversa	4	15	6.00	5	90	0	20	0	9	6.949	1.03	0.580	-0.14	1.121	0.22388	>0.20	<0.05
H. confusa	1	15	1.33	0	20	0	7	0	1	2.320	1.71	0.580	1.62	1.121	0.35713	<0.05	<0.01
H. confusa	2	15	1.40	1	21	0	9	0	2	2.324	2.79	0.580	8.93	1.121	0.27343	<0.20	<0.01
H. confusa	3	15	0.47	0	7	0	3	0	1	0.915	2.05	0.580	3.65	1.121	0.42822	<0.01	<0.01
H. confusa	4	15	2.13	1	32	0	8	0	4	2.475	1.06	0.580	0.51	1.121	0.20985	>0.20	<0.10
H. trivittata	1	15	0.53	0	8	0	2	0	1	0.834	1.16	0.580	-0.41	1.121	0.40546	<0.01	<0.01
H. trivittata	2	15	0.80	0	12	0	4	0	2	1.320	1.50	0.580	1.17	1.121	0.39440	<0.05	<0.01
H. trivittata	3	15	0.00	0	0	0	0	0	0	0.000			0.00				
H. trivittata	4	15	1.40	1	21	0	5	0	3	1.724	1.11	0.580	-0.17	1.121	0.32508	<0.10	<0.01
M. poecilogastra	1	15	0.00	0	0	0	0	0	0	0.000			0.00				

M. poecilogastra	2	15	10.00	4	150	0	39	1	20	12.095	1.33	0.580	0.84	1.121	0.26034	>0.20	<0.01
M. poecilogastra	3	15	0.00	0	0	0	0	0	0	0.000			0.00				
M. poecilogastra	4	15	3.40	2	51	0	16	1	5	4.188	2.17	0.580	5.59	1.121	0.21790	>0.20	<0.10
S. rufifrons	1	15	27.33	18	410	0	108	2	54	31.185	1.43	0.580	1.82	1.121	0.22192	>0.20	<0.05
S. rufifrons	2	15	16.67	9	250	0	61	2	29	19.609	1.29	0.580	0.76	1.121	0.19768	>0.20	<0.15
S. rufifrons	3	15	3.40	2	51	0	12	0	6	3.814	0.96	0.580	0.12	1.121	0.21369	>0.20	<0.10
S. rufifrons	4	15	15.27	12	229	0	39	2	26	13.729	0.49	0.580	-1.16	1.121	0.16836	>0.20	<0.01
S. graminum	1	15	0.80	0	12	0	4	0	2	1.207	1.56	0.580	2.17	1.121	0.34625	<0.05	<0.01
S. graminum	2	15	0.00	0	0	0	0	0	0	0.000			0.00				
S. graminum	3	15	0.27	0	4	0	2	0	0	0.594	2.27	0.580	4.78	1.121	0.47336	<0.01	<0.01
S. graminum	4	15	0.00	0	0	0	0	0	0	0.000			0.00				
S. pallida	1	15	3.93	1	59	0	16	0	8	4.891	1.21	0.580	0.98	1.121	0.25899	>0.20	<0.01
S. pallida	2	15	0.20	0	3	0	1	0	0	0.414	1.67	0.580	0.90	1.121	0.48547	<0.01	<0.01
S. pallida	3	15	1.47	0	22	0	9	0	3	2.669	1.99	0.580	3.74	1.121	0.37534	<0.05	<0.01
S. pallida	4	15	0.40	0	6	0	2	0	0	0.828	1.67	0.580	0.90	1.121	0.48547	<0.01	<0.01

Site 1—1, Site 2—2, Site 3—3, Site 4—4. Lower Q—lower quartile, Upper Q—upper quartile. Std. Dev.—standard deviation. Min—minimum values in the sample. Max—maximum values in the sample. Std. Err.—standard error. K-S *d*—*D*-statistic of the Kolmogorov–Smirnov one-sample test for normality. K-S *p*—probability of normal sample distribution according to *D*-statistics. Lilliefors *p*—probability of normal sample distribution according to Lilliefors test.

Table S2. Descriptive statistics for samples pooled by Drosophila collection years.

Species	Year	Valid N	Mean	Median	Sum	Min	Max	Lower Q	Upper Q	Std. Dev.	Skewness	Std. Err.	Kurtosis	Std. Err.	K-S *d*	K-S *p*	Lilliefors *p*
A. albilabris	2021	20	0.00	0.0	0	0	0	0	0	0			0.00				
A. albilabris	2022	20	0.25	0.0	5	0	2	0	0	0.64	2.44	0.512	4.77	0.992	0.50226	<0.01	<0.01
A. albilabris	2023	20	0.20	0.0	4	0	2	0	0	0.52	2.74	0.512	7.40	0.992	0.49888	<0.01	<0.01
G. distigma	2021	20	0.10	0.0	2	0	1	0	0	0.31	2.89	0.512	7.04	0.992	0.52737	<0.01	<0.01
G. distigma	2022	20	0.45	0.0	9	0	2	0	1	0.60	1.00	0.512	0.19	0.992	0.37157	<0.01	<0.01
G. distigma	2023	20	0.25	0.0	5	0	3	0	0	0.72	3.40	0.512	12.34	0.992	0.48645	<0.01	<0.01

L. maculata	2021	20	0.05	0.0	1	0	1	0	0	0.22	4.47	0.512	20.00	0.992	0.53847	<0.01	<0.01
L. maculata	2022	20	0.30	0.0	6	0	3	0	0	0.73	3.02	0.512	9.99	0.992	0.45889	<0.01	<0.01
L. maculata	2023	20	0.05	0.0	1	0	1	0	0	0.22	4.47	0.512	20.00	0.992	0.53847	<0.01	<0.01
L. quinquemaculata	2021	20	4.20	4.0	84	0	13	0	8	4.23	0.63	0.512	-0.78	0.992	0.18990	>0.20	<0.10
L. quinquemaculata	2022	20	5.60	5.5	112	0	14	2	9	4.25	0.34	0.512	-0.92	0.992	0.15165	>0.20	>0.20
L. quinquemaculata	2023	20	3.75	3.0	75	0	11	1	7	3.71	0.72	0.512	-0.83	0.992	0.18007	>0.20	<0.10
P. semivirgo	2021	20	4.30	1.0	86	0	20	0	7	6.74	1.60	0.512	1.28	0.992	0.28789	<0.10	<0.01
P. semivirgo	2022	20	6.75	1.5	135	0	31	0	11	9.77	1.48	0.512	1.16	0.992	0.26088	<0.15	<0.01
P. semivirgo	2023	20	5.15	1.0	103	0	24	0	8	7.75	1.53	0.512	1.04	0.992	0.30921	<0.05	<0.01
C. amoena	2021	20	0.25	0.0	5	0	2	0	0	0.55	2.24	0.512	4.66	0.992	0.47525	<0.01	<0.01
C. amoena	2022	20	0.90	0.0	18	0	5	0	2	1.37	1.69	0.512	2.89	0.992	0.34398	<0.05	<0.01
C. amoena	2023	20	0.20	0.0	4	0	1	0	0	0.41	1.62	0.512	0.70	0.992	0.48699	<0.01	<0.01
C. fuscimana	2021	20	0.45	0.0	9	0	2	0	1	0.69	1.28	0.512	0.54	0.992	0.39398	<0.01	<0.01
C. fuscimana	2022	20	0.35	0.0	7	0	2	0	1	0.67	1.78	0.512	2.02	0.992	0.44908	<0.01	<0.01
C. fuscimana	2023	20	0.35	0.0	7	0	2	0	1	0.59	1.52	0.512	1.64	0.992	0.42445	<0.01	<0.01
D. bifasciata	2021	20	0.60	0.0	12	0	3	0	1	0.94	1.37	0.512	0.75	0.992	0.38829	<0.01	<0.01
D. bifasciata	2022	20	0.45	0.0	9	0	2	0	1	0.69	1.28	0.512	0.54	0.992	0.39398	<0.01	<0.01
D. bifasciata	2023	20	0.55	0.0	11	0	2	0	1	0.83	1.07	0.512	-0.59	0.992	0.39736	<0.01	<0.01
D. busckii	2021	20	4.50	0.0	90	0	41	0	2	10.97	2.76	0.512	7.15	0.992	0.37514	<0.01	<0.01
D. busckii	2022	20	4.65	0.0	93	0	35	0	3	10.52	2.41	0.512	4.88	0.992	0.42077	<0.01	<0.01
D. busckii	2023	20	2.25	0.0	45	0	25	0	0	6.56	3.07	0.512	8.77	0.992	0.43419	<0.01	<0.01
D. funebris	2021	20	1.05	0.0	21	0	8	0	2	1.88	2.94	0.512	10.21	0.992	0.28796	<0.10	<0.01
D. funebris	2022	20	1.20	0.5	24	0	7	0	2	1.88	2.11	0.512	4.39	0.992	0.29235	<0.10	<0.01
D. funebris	2023	20	0.50	0.0	10	0	2	0	1	0.76	1.19	0.512	-0.04	0.992	0.39445	<0.01	<0.01
D. histrio	2021	20	11.65	5.5	233	0	55	0	18	14.90	1.62	0.512	2.57	0.992	0.21710	>0.20	<0.05
D. histrio	2022	20	18.75	4.5	375	0	124	1	18	32.52	2.53	0.512	6.14	0.992	0.31019	<0.05	<0.01
D. histrio	2023	20	10.65	7.5	213	0	31	0	20	11.02	0.43	0.512	-1.36	0.992	0.23302	<0.20	<0.01

D. hydei	2021	20	0.10	0.0	2	0	1	0	0	0.31	2.89	0.512	7.04	0.992	0.52737	<0.01	<0.01
D. hydei	2022	20	0.30	0.0	6	0	3	0	0	0.80	2.78	0.512	7.30	0.992	0.49594	<0.01	<0.01
D. hydei	2023	20	0.00	0.0	0	0	0	0	0	0.00			0.00				
D. immigrans	2021	20	4.90	0.0	98	0	52	0	3	12.97	3.18	0.512	10.08	0.992	0.37767	<0.01	<0.01
D. immigrans	2022	20	6.15	0.0	123	0	49	0	3	14.34	2.60	0.512	5.78	0.992	0.40959	<0.01	<0.01
D. immigrans	2023	20	4.40	0.0	88	0	41	0	3	11.33	2.86	0.512	7.27	0.992	0.37889	<0.01	<0.01
D. kuntzei	2021	20	8.90	0.0	178	0	76	0	5	20.19	2.59	0.512	6.37	0.992	0.42660	<0.01	<0.01
D. kuntzei	2022	20	15.95	0.5	319	0	121	0	4	36.09	2.26	0.512	3.85	0.992	0.41922	<0.01	<0.01
D. kuntzei	2023	20	6.10	0.0	122	0	50	0	2	14.16	2.41	0.512	4.94	0.992	0.41389	<0.01	<0.01
D. melanogaster	2021	20	21.35	2.0	427	0	140	0	23	38.28	2.18	0.512	4.43	0.992	0.31534	<0.05	<0.01
D. melanogaster	2022	20	22.95	6.5	459	0	131	0	38	35.02	1.98	0.512	3.82	0.992	0.32273	<0.05	<0.01
D. melanogaster	2023	20	44.10	24.5	882	0	234	1	50	67.36	2.16	0.512	4.16	0.992	0.25670	<0.15	<0.01
D. obscura	2021	20	175.75	177.5	3515	56	351	95	240	92.51	0.43	0.512	-1.03	0.992	0.14776	>0.20	>0.20
D. obscura	2022	20	197.30	186.0	3946	45	412	100	282	110.48	0.39	0.512	-0.97	0.992	0.13265	>0.20	>0.20
D. obscura	2023	20	182.10	200.5	3642	60	373	97	257	94.00	0.36	0.512	-1.02	0.992	0.21887	>0.20	<0.05
D. phalerata	2021	20	53.55	39.0	1071	0	160	4	90	53.03	0.75	0.512	-0.58	0.992	0.16506	>0.20	<0.20
D. phalerata	2022	20	79.35	19.5	1587	0	521	4	112	131.18	2.49	0.512	6.61	0.992	0.27262	<0.10	<0.01
D. phalerata	2023	20	51.10	44.5	1022	0	144	4	87	47.90	0.56	0.512	-0.93	0.992	0.16026	>0 .20	<0.20
D. repleta	2021	20	21.65	0.0	433	0	203	0	5	53.43	2.69	0.512	6.99	0.992	0.43630	<0.01	<0.01
D. repleta	2022	20	13.15	0.0	263	0	108	0	4	31.02	2.37	0.512	4.59	0.992	0.42858	<0.01	<0.01
D. repleta	2023	20	4.50	0.0	90	0	43	0	0	11.13	2.81	0.512	7.87	0.992	0.45703	<0.01	<0.01
D. subobscura	2021	20	0.15	0.0	3	0	1	0	0	0.37	2.12	0.512	2.78	0.992	0.50889	<0.01	<0.01
D. subobscura	2022	20	0.65	0.0	13	0	5	0	1	1.31	2.45	0.512	6.17	0.992	0.39027	<0.01	<0.01
D. subobscura	2023	20	0.60	0.0	12	0	3	0	1	0.94	1.37	0.512	0.75	0.992	0.38829	<0.01	<0.01
D. subsilvestris	2021	20	7.00	2.5	140	0	27	0	12	8.70	1.21	0.512	0.32	0.992	0.22717	>0.20	<0.05
D. subsilvestris	2022	20	8.75	3.5	175	0	33	1	14	10.35	1.15	0.512	0.23	0.992	0.22692	>0.20	<0.05
D. subsilvestris	2023	20	7.80	3.0	156	0	30	1	13	9.41	1.21	0.512	0.30	0.992	0.26695	<0.10	<0.01

D. testacea	2021	20	70.05	60.5	1401	0	255	2	117	74.65	0.99	0.512	0.55	0.992	0.21187	>0.20	<0.05
D. testacea	2022	20	56.10	63.5	1122	0	203	1	91	55.81	0.87	0.512	0.82	0.992	0.21534	>0.20	<0.05
D. testacea	2023	20	69.00	69.5	1380	0	308	1	101	76.85	1.62	0.512	3.82	0.992	0.19751	>0.20	<0.05
D. transversa	2021	20	7.65	6.5	153	0	21	2	13	6.66	0.62	0.512	-0.52	0.992	0.13888	>0.20	>0.20
D. transversa	2022	20	13.40	12.0	268	0	41	3	19	12.23	0.80	0.512	-0.18	0.992	0.15387	>0.20	>0.20
D. transversa	2023	20	6.55	5.5	131	0	18	1	10	5.79	0.55	0.512	-0.72	0.992	0.13111	>0.20	>0.20
H. confusa	2021	20	0.95	0.0	19	0	5	0	1	1.67	1.75	0.512	1.89	0.992	0.36535	<0.01	<0.01
H. confusa	2022	20	1.15	0.5	23	0	5	0	2	1.60	1.53	0.512	1.66	0.992	0.26406	<0.10	<0.01
H. confusa	2023	20	1.90	0.5	38	0	9	0	3	2.88	1.61	0.512	1.44	0.992	0.27259	<0.10	<0.01
H. trivittata	2021	20	0.55	0.0	11	0	4	0	1	1.05	2.28	0.512	5.49	0.992	0.39978	<0.01	<0.01
H. trivittata	2022	20	1.10	0.0	22	0	5	0	2	1.65	1.30	0.512	0.39	0.992	0.34736	<0.05	<0.01
H. trivittata	2023	20	0.40	0.0	8	0	3	0	1	0.82	2.26	0.512	4.90	0.992	0.43699	<0.01	<0.01
M. poecilogastra	2021	20	3.35	0.0	67	0	39	0	2	9.12	3.61	0.512	13.61	0.992	0.41531	<0.01	<0.01
M. poecilogastra	2022	20	3.70	0.0	74	0	25	0	4	7.06	2.29	0.512	4.66	0.992	0.30003	<0.05	<0.01
M. poecilogastra	2023	20	3.00	0.0	60	0	27	0	5	6.33	3.19	0.512	11.49	0.992	0.32393	<0.05	<0.01
S. rufifrons	2021	20	11.90	2.0	238	0	59	1	21	18.39	1.70	0.512	1.84	0.992	0.33582	<0.05	<0.01
S. rufifrons	2022	20	18.60	10.5	372	0	108	2	21	26.28	2.48	0.512	6.91	0.992	0.24853	<0.15	<0.01
S. rufifrons	2023	20	16.50	10.0	330	0	61	5	29	17.98	1.33	0.512	0.98	0.992	0.27717	<0.10	<0.01
S. graminum	2021	20	0.15	0.0	3	0	2	0	0	0.49	3.44	0.512	11.89	0.992	0.52040	<0.01	<0.01
S. graminum	2022	20	0.20	0.0	4	0	2	0	0	0.52	2.74	0.512	7.40	0.992	0.49888	<0.01	<0.01
S. graminum	2023	20	0.45	0.0	9	0	4	0	0	1.05	2.57	0.512	6.63	0.992	0.46587	<0.01	<0.01
S. pallida	2021	20	0.90	0.0	18	0	8	0	1	2.17	2.74	0.512	6.90	0.992	0.41056	<0.01	<0.01
S. pallida	2022	20	1.20	0.0	24	0	8	0	2	2.31	2.04	0.512	3.52	0.992	0.39845	<0.01	<0.01
S. pallida	2023	20	2.40	0.0	48	0	16	0	3	4.37	2.17	0.512	4.36	0.992	0.32566	<0.05	<0.01

Designations as in Table S1.

Table. S3. Descriptive statistics for samples pooled by Drosophila collection month.

Species	Month	Valid N	Mean	Median	Sum	Min	Max	Lower Q	Upper Q	Std. Dev.	Skewness	Std. Err.	Kurtosis	Std. Err.	K-S d	K-S p	Lilliefors p
A. albilabris	May	12	0.00	0	0	0	0	0	0	0.000			0.00				
A. albilabris	June	12	0.00	0	0	0	0	0	0	0.000			0.00				
A. albilabris	July	12	0.33	0	4	0	2	0	1	0.651	1.93	0.637	3.17	1.232	0.44559	<0.05	<0.01
A. albilabris	Aug.	12	0.42	0	5	0	2	0	1	0.793	1.64	0.637	1.13	1.232	0.45037	<0.01	<0.01
A. albilabris	Sept.	12	0.00	0	0	0	0	0	0	0.000			0.00				
G. distigma	May	12	0.00	0	0	0	0	0	0	0.000			0.00				
G. distigma	June	12	0.08	0	1	0	1	0	0	0.289	3.46	0.637	12.00	1.232	0.53025	<0.01	<0.01
G. distigma	July	12	0.17	0	2	0	1	0	0	0.389	2.06	0.637	2.64	1.232	0.49907	<0.01	<0.01
G. distigma	Aug.	12	0.75	1	9	0	3	0	1	0.965	1.32	0.637	1.41	1.232	0.28141	>0.20	<0.01
G. distigma	Sept.	12	0.33	0	4	0	1	0	1	0.492	0.81	0.637	-1.65	1.232	0.41746	<0.05	<0.01
L. maculata	May	12	0.00	0	0	0	0	0	0	0.000			0.00				
L. maculata	June	12	0.42	0	5	0	3	0	1	0.900	2.54	0.637	6.77	1.232	0.42824	<0.05	<0.01
L. maculata	July	12	0.17	0	2	0	1	0	0	0.389	2.06	0.637	2.64	1.232	0.49907	<0.01	<0.01
L. maculata	Aug.	12	0.00	0	0	0	0	0	0	0.000			0.00				
L. maculata	Sept.	12	0.08	0	1	0	1	0	0	0.289	3.46	0.637	12.00	1.232	0.53025	<0.01	<0.01
L. quinquemaculata	May	12	6.58	9	79	0	14	2	11	4.944	-0.24	0.637	-1.44	1.232	0.19609	>0.20	>0.20
L. quinquemaculata	June	12	3.50	4	42	0	6	2	5	1.977	-0.34	0.637	-1.01	1.232	0.19264	>0.20	>0.20
L. quinquemaculata	July	12	7.75	9	93	0	13	5	11	4.181	-0.82	0.637	-0.35	1.232	0.20086	>0.20	<0.20

L. quinquemaculata	Aug.	12	0.50	0	6	0	2	0	1	0.674	1.07	0.637	0.35	1.232	0.35417	<0.10	<0.01
L. quinquemaculata	Sept.	12	4.25	4	51	0	8	2	7	2.832	-0.33	0.637	-1.23	1.232	0.16753	>0.20	<0.20
P. semivirgo	May	12	0.00	0	0	0	0	0	0	0.000			0.00				
P. semivirgo	June	12	2.00	1	24	0	9	1	3	2.523	2.20	0.637	5.58	1.232	0.25000	>0.20	<0.05
P. semivirgo	July	12	7.50	8	90	0	14	5	11	4.739	-0.31	0.637	-0.71	1.232	0.12579	>0.20	>0.20
P. semivirgo	Aug.	12	16.92	20	203	0	31	10	23	10.184	-0.74	0.637	-0.56	1.232	0.25326	>0.20	<0.05
P. semivirgo	Sept.	12	0.58	0	7	0	3	0	1	0.900	1.95	0.637	4.37	1.232	0.32481	<0.15	<0.01
C. amoena	May	12	0.00	0	0	0	0	0	0	0.000			0.00				
C. amoena	June	12	0.17	0	2	0	2	0	0	0.577	3.46	0.637	12.00	1.232	0.53025	<0.01	<0.01
C. amoena	July	12	1.00	1	12	0	3	0	2	1.044	0.57	0.637	-0.86	1.232	0.24749	>0.20	<0.05
C. amoena	Aug.	12	0.58	1	7	0	2	0	1	0.669	0.74	0.637	-0.19	1.232	0.30854	<0.20	<0.01
C. amoena	Sept.	12	0.50	0	6	0	5	0	0	1.446	3.25	0.637	10.77	1.232	0.46858	<0.01	<0.01
C. fuscimana	May	12	0.75	1	9	0	2	0	1	0.622	0.17	0.637	-0.09	1.232	0.32290	<0.15	<0.01
C. fuscimana	June	12	1.17	1	14	0	2	1	2	0.718	-0.26	0.637	-0.69	1.232	0.25848	>0.20	<0.05
C. fuscimana	July	12	0.00	0	0	0	0	0	0	0.000			0.00				
C. fuscimana	Aug.	12	0.00	0	0	0	0	0	0	0.000			0.00				
C. fuscimana	Sept.	12	0.00	0	0	0	0	0	0	0.000			0.00				
D. bifasciata	May	12	0.08	0	1	0	1	0	0	0.289	3.46	0.637	12.00	1.232	0.53025	<0.01	<0.01
D. bifasciata	June	12	1.17	1	14	0	2	1	2	0.835	-0.35	0.637	-1.45	1.232	0.25757	>0.20	<0.05
D. bifasciata	July	12	1.42	1	17	0	3	1	2	0.793	0.33	0.637	0.33	1.232	0.28370	>0.20	<0.01
D. bifasciata	Aug.	12	0.00	0	0	0	0	0	0	0.000			0.00				
D. bifasciata	Sept.	12	0.00	0	0	0	0	0	0	0.000			0.00				
D. busckii	May	12	0.00	0	0	0	0	0	0	0.000			0.00				
D. busckii	June	12	0.33	0	4	0	4	0	0	1.155	3.46	0.637	12.00	1.232	0.53025	<0.01	<0.01
D. busckii	July	12	5.25	0	63	0	29	0	8	9.659	1.79	0.637	2.34	1.232	0.42003	<0.05	<0.01
D. busckii	Aug.	12	9.75	1	117	0	41	0	19	14.654	1.35	0.637	0.45	1.232	0.29375	>0.20	<0.01

D. busckii	Sept.	12	3.67	0	44	0	35	0	2	10.039	3.27	0.637	10.94	1.232	0.39253	<0.05	<0.01
D. funebris	May	12	0.00	0	0	0	0	0	0	0.000			0.00				
D. funebris	June	12	0.50	0	6	0	2	0	1	0.674	1.07	0.637	0.35	1.232	0.35417	<0.10	<0.01
D. funebris	July	12	2.42	2	29	0	8	1	3	2.539	1.56	0.637	1.63	1.232	0.31517	<0.15	<0.01
D. funebris	Aug.	12	1.08	1	13	0	3	0	2	0.996	0.47	0.637	-0.65	1.232	0.20000	>0.20	<0.20
D. funebris	Sept.	12	0.58	0	7	0	5	0	1	1.443	3.07	0.637	9.82	1.232	0.40695	<0.05	<0.01
D. histrio	May	12	0.42	0	5	0	4	0	0	1.165	3.14	0.637	10.13	1.232	0.47309	<0.01	<0.01
D. histrio	June	12	13.42	14	161	0	44	7	16	11.309	1.78	0.637	4.99	1.232	0.25931	>0.20	<0.05
D. histrio	July	12	3.08	3	37	0	9	1	6	3.029	0.75	0.637	-0.56	1.232	0.17764	>0.20	>0.20
D. histrio	Aug.	12	20.67	18	248	0	90	8	22	23.815	2.49	0.637	7.54	1.232	0.31101	<0.20	<0.01
D. histrio	Sept.	12	30.83	24	370	0	124	12	34	33.108	2.25	0.637	6.16	1.232	0.27133	>0.20	<0.05
D. hydei	May	12	0.00	0	0	0	0	0	0	0.000			0.00				
D. hydei	June	12	0.08	0	1	0	1	0	0	0.289	3.46	0.637	12.00	1.232	0.53025	<0.01	<0.01
D. hydei	July	12	0.17	0	2	0	1	0	0	0.389	2.06	0.637	2.64	1.232	0.49907	<0.01	<0.01
D. hydei	Aug.	12	0.17	0	2	0	2	0	0	0.577	3.46	0.637	12.00	1.232	0.53025	<0.01	<0.01
D. hydei	Sept.	12	0.25	0	3	0	3	0	0	0.866	3.46	0.637	12.00	1.232	0.53025	<0.01	<0.01
D. immigrans	May	12	0.00	0	0	0	0	0	0	0.000			0.00				
D. immigrans	June	12	0.00	0	0	0	0	0	0	0.000			0.00				
D. immigrans	July	12	2.83	1	34	0	17	0	4	4.933	2.49	0.637	6.82	1.232	0.28286	>0.20	<0.01
D. immigrans	Aug.	12	9.83	2	118	0	44	0	17	15.948	1.46	0.637	0.53	1.232	0.39273	<0.05	<0.01
D. immigrans	Sept.	12	13.08	4	157	0	52	0	23	20.865	1.35	0.637	-0.09	1.232	0.41834	<0.05	<0.01
D. kuntzei	May	12	0.08	0	1	0	1	0	0	0.289	3.46	0.637	12.00	1.232	0.53025	<0.01	<0.01
D. kuntzei	June	12	13.58	0	163	0	91	0	14	28.318	2.28	0.637	5.09	1.232	0.43427	<0.05	<0.01
D. kuntzei	July	12	13.17	0	158	0	82	0	19	25.915	2.09	0.637	4.13	1.232	0.41673	<0.05	<0.01
D. kuntzei	Aug.	12	2.42	1	29	0	12	0	4	3.502	2.16	0.637	5.05	1.232	0.29735	<0.20	<0.01
D. kuntzei	Sept.	12	22.33	4	268	0	121	1	28	39.334	1.90	0.637	2.89	1.232	0.42027	<0.05	<0.01
D. melanogaster	May	12	0.00	0	0	0	0	0	0	0.000			0.00				
D. melanogaster	June	12	2.58	3	31	0	6	0	5	2.575	0.25	0.637	-1.74	1.232	0.25883	>0.20	<0.05
D. melanogaster	July	12	14.08	8	169	0	41	2	30	15.436	0.82	0.637	-1.17	1.232	0.29571	<0.20	<0.01
D. melanogaster	Aug.	12	83.58	47	1003	4	234	32	121	76.650	1.11	0.637	0.10	1.232	0.26209	>0.20	<0.05

D. melanogaster	Sept.	12	47.08	53	565	1	131	15	68	37.761	0.77	0.637	0.71	1.232	0.17180	>0.20	>0.20
D. obscura	May	12	85.17	80	1022	45	148	68	99	27.742	0.86	0.637	1.29	1.232	0.12877	>0.20	>0.20
D. obscura	June	12	234.33	238	2812	170	316	197	265	43.297	0.25	0.637	-0.47	1.232	0.09870	>0.20	>0.20
D. obscura	July	12	326.50	320	3918	279	412	290	362	43.444	0.76	0.637	-0.47	1.232	0.21704	>0.20	<0.15
D. obscura	Aug.	12	96.83	98	1162	57	133	79	111	24.461	-0.27	0.637	-0.68	1.232	0.15308	>0.20	>0.20
D. obscura	Sept.	12	182.42	201	2189	98	249	143	213	50.832	-0.75	0.637	-0.67	1.232	0.21819	>0.20	<0.15
D. phalerata	May	12	0.75	0	9	0	5	0	1	1.603	2.24	0.637	4.44	1.232	0.43011	<0.05	<0.01
D. phalerata	June	12	117.92	92	1415	14	521	28	141	137.076	2.62	0.637	7.91	1.232	0.29609	<0.20	<0.01
D. phalerata	July	12	35.50	32	426	2	118	7	52	32.856	1.41	0.637	2.82	1.232	0.15396	>0.20	>0.20
D. phalerata	Aug.	12	54.50	45	654	1	174	15	84	49.061	1.30	0.637	2.16	1.232	0.17886	>0.20	>0.20
D. phalerata	Sept.	12	98.00	99	1176	5	316	31	133	86.089	1.39	0.637	3.12	1.232	0.18191	>0.20	>0.20
D. repleta	May	12	0.00	0	0	0	0	0	0	0.000			0.00				
D. repleta	June	12	0.00	0	0	0	0	0	0	0.000			0.00				
D. repleta	July	12	17.42	2	209	0	96	0	18	31.126	2.02	0.637	3.33	1.232	0.34417	<0.10	<0.01
D. repleta	Aug.	12	21.58	2	259	0	114	0	28	37.306	1.87	0.637	2.75	1.232	0.34099	<0.10	<0.01
D. repleta	Sept.	12	26.50	0	318	0	203	0	4	63.600	2.49	0.637	5.78	1.232	0.45376	<0.01	<0.01
D. subobscura	May	12	0.00	0	0	0	0	0	0	0.000			0.00				
D. subobscura	June	12	0.67	1	8	0	2	0	1	0.778	0.72	0.637	-0.79	1.232	0.30410	<0.20	<0.01
D. subobscura	July	12	1.08	1	13	0	3	0	2	1.165	0.64	0.637	-1.01	1.232	0.24056	>0.20	<0.10
D. subobscura	Aug.	12	0.17	0	2	0	1	0	0	0.389	2.06	0.637	2.64	1.232	0.49907	<0.01	<0.01
D. subobscura	Sept.	12	0.42	0	5	0	5	0	0	1.443	3.46	0.637	12.00	1.232	0.53025	<0.01	<0.01
D. subsilvestris	May	12	0.00	0	0	0	0	0	0	0.000			0.00				
D. subsilvestris	June	12	1.50	1	18	0	4	1	2	1.168	0.82	0.637	0.61	1.232	0.24907	>0.20	<0.05
D. subsilvestris	July	12	5.75	5	69	2	12	3	8	3.108	0.78	0.637	-0.21	1.232	0.17868	>0.20	>0.20
D. subsilvestris	Aug.	12	20.92	24	251	2	33	17	28	10.040	-1.08	0.637	0.15	1.232	0.25331	>0.20	<0.05
D. subsilvestris	Sept.	12	11.08	13	133	0	22	7	15	6.775	-0.52	0.637	-0.43	1.232	0.24509	>0.20	<0.05
D. testacea	May	12	164.17	139	1970	94	308	115	206	66.994	1.05	0.637	0.35	1.232	0.20164	>0.20	<0.20
D. testacea	June	12	89.50	84	1074	67	121	75	103	18.367	0.58	0.637	-0.86	1.232	0.18010	>0.20	>0.20
D. testacea	July	12	1.58	1	19	0	6	0	2	1.832	1.49	0.637	2.13	1.232	0.24337	>0.20	<0.05
D. testacea	Aug.	12	1.08	1	13	0	5	0	1	1.379	2.32	0.637	6.49	1.232	0.35743	<0.10	<0.01

D. testacea	Sept.	12	68.92	68	827	40	95	56	89	18.749	-0.08	0.637	-1.26	1.232	0.17895	>0.20	>0.20
D. transversa	May	12	0.25	0	3	0	2	0	0	0.622	2.56	0.637	6.24	1.232	0.48956	<0.01	<0.01
D. transversa	June	12	20.67	18	248	7	41	16	25	9.374	1.05	0.637	0.95	1.232	0.23582	>0.20	<0.10
D. transversa	July	12	12.25	11	147	5	30	8	15	7.098	1.54	0.637	2.77	1.232	0.18256	>0.20	>0.20
D. transversa	Aug.	12	8.33	8	100	4	18	5	10	4.271	1.19	0.637	1.08	1.232	0.18798	>0.20	>0.20
D. transversa	Sept.	12	4.50	4	54	0	14	1	6	4.400	1.05	0.637	0.73	1.232	0.19993	>0.20	<0.20
H. confusa	May	12	4.42	5	53	0	9	3	6	2.746	0.08	0.637	-0.68	1.232	0.16746	>0.20	>0.20
H. confusa	June	12	0.00	0	0	0	0	0	0	0.000			0.00				
H. confusa	July	12	0.00	0	0	0	0	0	0	0.000			0.00				
H. confusa	Aug.	12	0.67	1	8	0	2	0	1	0.778	0.72	0.637	-0.79	1.232	0.30410	<0.20	<0.01
H. confusa	Sept.	12	1.58	1	19	0	4	1	3	1.240	0.63	0.637	-0.34	1.232	0.26429	>0.20	<0.05
H. trivittata	May	12	0.08	0	1	0	1	0	0	0.289	3.46	0.637	12.00	1.232	0.53025	<0.01	<0.01
H. trivittata	June	12	1.17	1	14	0	4	0	2	1.337	1.01	0.637	0.20	1.232	0.22521	>0.20	<0.10
H. trivittata	July	12	0.17	0	2	0	1	0	0	0.389	2.06	0.637	2.64	1.232	0.49907	<0.01	<0.01
H. trivittata	Aug.	12	0.00	0	0	0	0	0	0	0.000			0.00				
H. trivittata	Sept.	12	2.00	2	24	0	5	1	4	1.706	0.40	0.637	-0.98	1.232	0.16667	>0.20	>0.20
M. poecilogastra	May	12	0.00	0	0	0	0	0	0	0.000			0.00				
M. poecilogastra	June	12	3.25	1	39	0	20	0	4	5.910	2.47	0.637	6.36	1.232	0.29120	>0.20	<0.01
M. poecilogastra	July	12	2.08	1	25	0	11	0	4	3.232	2.16	0.637	5.30	1.232	0.25961	>0.20	<0.05
M. poecilogastra	Aug.	12	1.67	1	20	0	8	0	3	2.605	1.70	0.637	2.15	1.232	0.35098	<0.10	<0.01
M. poecilogastra	Sept.	12	9.75	3	117	0	39	0	21	13.619	1.22	0.637	0.23	1.232	0.30304	<0.20	<0.01
S. rufifrons	May	12	0.25	0	3	0	2	0	0	0.622	2.56	0.637	6.24	1.232	0.48956	<0.01	<0.01
S. rufifrons	June	12	8.17	7	98	0	22	2	13	7.396	0.64	0.637	-0.67	1.232	0.17427	>0.20	>0.20
S. rufifrons	July	12	22.67	21	272	2	64	14	29	16.445	1.32	0.637	3.10	1.232	0.16968	>0.20	>0.20
S. rufifrons	Aug.	12	41.50	37	498	6	108	21	57	28.548	0.92	0.637	1.57	1.232	0.16395	>0.20	>0.20
S. rufifrons	Sept.	12	5.75	5	69	0	18	2	8	5.294	1.33	0.637	1.46	1.232	0.22300	>0.20	<0.15
S. graminum	May	12	0.00	0	0	0	0	0	0	0.000			0.00				
S. graminum	June	12	0.00	0	0	0	0	0	0	0.000			0.00				
S. graminum	July	12	0.83	0	10	0	4	0	2	1.337	1.46	0.637	1.39	1.232	0.40010	<0.05	<0.01
S. graminum	Aug.	12	0.50	0	6	0	2	0	1	0.674	1.07	0.637	0.35	1.232	0.35417	<0.10	<0.01

S. graminum	Sept.	12	0.00	0	0	0	0	0	0	0.000			0.00				
S. pallida	May	12	0.00	0	0	0	0	0	0	0.000			0.00				
S. pallida	June	12	0.00	0	0	0	0	0	0	0.000			0.00				
S. pallida	July	12	2.50	0	30	0	10	0	6	3.451	1.10	0.637	0.16	1.232	0.34893	<0.10	<0.01
S. pallida	Aug.	12	4.33	2	52	0	16	1	8	4.942	1.33	0.637	1.42	1.232	0.26492	>0.20	<0.05
S. pallida	Sept.	12	0.67	1	8	0	3	0	1	0.888	1.73	0.637	3.81	1.232	0.27369	>0.20	<0.05

Designations as in Table S1. Aug.—August, Sept.—September.

Table S4. The influence of the “Year” factor on the abundance of species in collections from city parks.

Species	Bitsevsky Park		Fili Park		The Main Botanical Garden		Suvorovsky Park	
	K-W p	Med. p	K-W p	Med. p	K-W p	Med. p	K-W p	Med. p
A. albilabris	1	1	0.5836	0.5616	1	1	0.2818	0.2557
G. distigma	0.4999	0.4346	0.3679	0.3425	0.116	0.0995	0.7111	1
L. maculata	1	1	0.3679	0.3425	1	1	0.2671	0.3012
L..quinquemaculata	0.1612	0.0821	0.8313	0.765	0.1163	0.1225	0.8858	0.765
P..semivirgo	0.7604	0.765	0.827	0.765	0.3389	0.2865	0.5993	0.765
C. amoena	1	1	0.546	0.765	0.6376	0.7111	0.1643	0.3012
C..fuscimana	0.9608	1	0.857	0.7408	0.116	0.0995	0.857	0.7408
D..bifasciata	0.8646	0.765	0.799	0.7408	0.5859	0.7408	0.7275	0.7111
D. busckii	1	1	0.5517	0.765	1	1	0.9352	1
D. funebris	0.9847	1	0.2671	0.3012	0.6214	0.765	0.1853	0.0357
D. histrio	0.6167	0.765	0.7712	0.765	0.1698	0.1225	0.7909	0.765
D. hydei	1	1	0.3679	0.3425	1	1	0.0878	0.092
D..immigrans	0.2662	0.2865	0.7919	0.765	1	1	0.7037	0.3425
D. kuntzei	0.3778	0.3425	0.6376	0.7111	0.286	0.2557	0.6095	0.7408
D..melanogaster	0.5756	0.765	0.783	0.765	0.7086	0.765	0.6118	0.765
D. obscura	0.6505	0.765	0.8264	0.765	0.8869	0.765	0.6126	0.765
D. phalerata	0.2699	0.765	0.9113	0.765	0.3078	0.3425	0.8445	1
D. repleta	1	1	0.3184	0.3425	1	1	0.9912	0.765
D..subobscura	0.9847	1	0.296	0.4346	1	1	0.2761	0.3425

D..subsilvestris	0.8862	0.765	0.9314	0.765	0.3719	0.2557	0.9774	1
D. testacea	0.9319	0.765	0.7318	0.765	0.9694	0.765	0.9123	0.765
D..transversa	0.3288	0.4346	0.3037	0.1534	0.4603	0.3425	0.9275	0.7408
H. confusa	0.9904	1	0.7889	0.3012	0.2712	0.2557	0.9301	0.765
H. trivittata	0.6204	0.7408	0.6505	0.7408	1	1	0.4157	0.7111
M..poecilogastra	1	1	0.6023	0.3425	1	1	0.9923	0.765
S. rufifrons	0.5227	0.765	0.8509	0.765	0.23	0.1534	0.8536	0.765
S..graminum	0.9168	1	1	1	0.2906	0.2865	1	1
S. pallida	0.8178	0.765	0.3114	0.2865	0.1379	0.1225	1	1
B-H 0.05	--	--	--	--	--	--	--	--

K-W test—Kruskal–Wallis ANOVA by rank. Med.—median test. B-H 0.05—Benjamini and Hochberg corrected values for the 0.05 level of significance. None of the multiple tests yielded significant results.

Table S5. The influence of the "Site" factor on the abundance of species in collections from city parks.

Species	2021		2022		2023	
	K-W p	Med. p	K-W p	Med. p	K-W p	Med. p
A. albilabris	1	1	0.2214	0.2295	0.2377	0.2295
G. distigma	0.5497	0.5276	0.8264	0.6444	0.2377	0.2295
L. maculata	0.3916	0.3679	0.0629	0.0576	0.3916	0.3679
L..quinquemaculata	0.0212	0.0528	0.0559	0.0658	0.0355	0.0396
P..semivirgo	0.368	0.6444	0.0869	0.0658	0.4327	0.6444
C. amoena	0.4565	0.4753	0.1708	0.1718	0.4983	0.4753
C..fuscimana	0.9536	0.8827	0.4756	0.402	0.4368	0.4142
D..bifasciata	0.8033	0.8827	0.5432	0.4904	0.795	0.8827
D. busckii	0.016	0.0149	0.0593	0.0658	0.1894	0.1718
D. funebris	0.1414	0.1414	0.0754	0.2615	0.6289	0.4904
D. histrio	0.1761	0.0658	0.0507	0.0658	0.0605	0.0658
D. hydei	0.5497	0.5275	0.0187	0.0142	1	1
D..immigrans	0.0482	0.0356	0.1418	0.1718	0.1923	0.1901
D. kuntzei	0.0726	0.2795	0.0158	0.0658	0.0222	0.0528
D..melanogaster	0.7859	0.895	0.3469	0.4936	0.5531	0.8495

D. obscura	0.7298	0.8495	0.4436	0.4936	0.6681	0.8495
D. phalerata	0.1536	0.0658	0.0259	0.0321	0.1391	0.0321
D. repleta	0.0591	0.0658	0.0426	0.0356	0.1894	0.1718
D..subobscura	0.7728	0.7587	0.214	0.1901	0.1091	0.1149
D..subsilvestris	0.2214	0.0658	0.3314	0.0658	0.7583	0.5276
D. testacea	0.5283	0.8495	0.6875	0.8495	0.6708	0.8495
D..transversa	0.7361	0.8495	0.2031	0.2615	0.4896	0.2615
H. confusa	0.2673	0.2431	0.6454	0.8495	0.7999	0.8495
H. trivittata	0.4516	0.4142	0.1233	0.0833	0.1925	0.167
M..poecilogastra	0.0085	0.004	0.0067	0.004	0.0075	0.004
S. rufifrons	0.6134	0.5276	0.0504	0.0658	0.5809	0.2615
S..graminum	0.0972	0.0833	0.2377	0.2295	0.1921	0.1718
S. pallida	0.1491	0.167	0.179	0.1901	0.3105	0.5276
B-H 0.05	--	--	--	--	--	--

K-W test—Kruskal–Wallis ANOVA by rank. Med.—median test. B-H 0.05—Benjamini and Hochberg corrected values for the 0.05 level of significance. None of the multiple tests yielded significant results.

Table S6. Influence of the "Year" factor on the abundance of species in monthly collections.

Species	May		June		July		August		September	
	K-W p	Med. p	K-W p	Med. p	K-W p	Med. p	K-W p	Med. p	K-W p	Med. p
A. albilabris	1	1	1	1	0.2703	0.2636	0.3301	0.2636	1	1
G. distigma	1	1	0.3679	0.3359	0.1108	0.0907	0.1413	0.0498	0.0902	0.0724
L. maculata	1	1	0.2703	0.2636	0.577	0.5488	1	1	0.3679	0.3359
L. quinquemaculata	0.5855	0.3679	0.4284	0.3679	0.8121	0.7097	0.219	0.2537	0.6365	0.2537
P. semivirgo	1	1	0.42	0.2537	0.4323	0.3679	0.4374	0.3679	0.6579	0.7097
C. amoena	1	1	0.3679	0.3359	0.2611	0.0724	0.9311	1	0.1128	0.0907
C. fuscimana	0.5662	0.3359	0.3037	0.2231	1	1	1	1	1	1
D. bifasciata	0.3679	0.3359	0.863	0.7097	0.1003	0.0907	1	1	1	1
D. busckii	1	1	0.3679	0.3359	0.7451	0.7097	0.9161	1	0.2406	0.2636
D. funebris	1	1	0.1028	0.0907	0.6302	0.2636	0.6203	0.6873	0.9753	1

D. histrio	0.1128	0.0907	0.5102	0.3679	0.603	0.3679	0.9952	0.3679	0.7788	0.3679
D. hydei	1	1	0.3679	0.3359	0.577	0.5488	0.3679	0.3359	0.3679	0.3359
D. immigrans	1	1	1	1	0.9364	1	0.9203	0.7097	0.9195	1
D. kuntzei	0.3679	0.3359	0.9674	1	0.9153	0.6873	0.7255	0.7097	0.2978	0.3679
D. melanogaster	1	1	0.1358	0.0498	0.0181	0.0183	0.1054	0.3679	0.2364	0.3679
D. obscura	0.8741	1	0.2757	0.3679	0.4176	0.3679	0.9808	1	0.7379	0.3679
D. phalerata	0.0279	0.0183	0.9439	1	0.8611	0.3679	0.926	1	0.8126	1
D. repleta	1	1	1	1	0.9364	1	0.9783	1	0.358	0.2636
D. subobscura	1	1	0.2154	0.3679	0.519	0.2231	0.1108	0.0907	0.3679	0.3359
D. subsilvestris	1	1	0.5631	0.2537	0.2419	0.0907	0.6831	1	0.7774	1
D. testacea	0.3679	0.3679	0.5114	0.3679	0.8187	0.7097	0.5369	0.5488	0.7351	1
D. transversa	0.1128	0.0907	0.0499	0.0907	0.5264	0.3679	0.3384	0.3679	0.6211	0.7097
H. confusa	0.1374	0.0183	1	1	1	1	0.3733	0.3679	0.6827	0.7097
H. trivittata	0.3679	0.3359	0.1344	0.0724	0.577	0.5488	1	1	0.6292	0.6873
M. poecilogastra	1	1	0.8578	1	0.8159	1	0.7487	1	0.931	1
S. rufifrons	0.1128	0.0907	0.0937	0.0498	0.4826	0.3679	0.8864	0.3679	0.1134	0.0498
S. graminum	1	1	1	1	0.594	0.6873	0.6579	0.7097	1	1
S. pallida	1	1	1	1	0.7725	0.7097	0.4032	0.7097	0.3059	0.3679
B-H 0.05	--	--	--	--	--	--	--	--	--	--

K-W test—Kruskal–Wallis ANOVA by rank. B-H 0.05—Benjamini and Hochberg corrected values for the 0.05 level of significance. None of the multiple tests yielded significant results.
